# Phylogenetics and environmental distribution of nitric oxide-forming nitrite reductases reveal their distinct functional and ecological roles

**DOI:** 10.1093/ismeco/ycae020

**Published:** 2024-02-02

**Authors:** Grace Pold, Germán Bonilla-Rosso, Aurélien Saghaï, Marc Strous, Christopher M Jones, Sara Hallin

**Affiliations:** Department of Forest Mycology and Plant Pathology, Swedish University of Agricultural Sciences, 750 07 Uppsala, Sweden; Department of Forest Mycology and Plant Pathology, Swedish University of Agricultural Sciences, 750 07 Uppsala, Sweden; Department of Forest Mycology and Plant Pathology, Swedish University of Agricultural Sciences, 750 07 Uppsala, Sweden; Department of Earth, Energy, and Environment, University of Calgary, Calgary, Alberta, T2N 1N4, Canada; Department of Forest Mycology and Plant Pathology, Swedish University of Agricultural Sciences, 750 07 Uppsala, Sweden; Department of Forest Mycology and Plant Pathology, Swedish University of Agricultural Sciences, 750 07 Uppsala, Sweden

**Keywords:** denitrification, nitrite reductase, metagenomes, comparative genomics, phylogenetics

## Abstract

The two evolutionarily unrelated nitric oxide-producing nitrite reductases, NirK and NirS, are best known for their redundant role in denitrification. They are also often found in organisms that do not perform denitrification. To assess the functional roles of the two enzymes and to address the sequence and structural variation within each, we reconstructed robust phylogenies of both proteins with sequences recovered from 6973 isolate and metagenome-assembled genomes and identified 32 well-supported clades of structurally distinct protein lineages. We then inferred the potential niche of each clade by considering other functional genes of the organisms carrying them as well as the relative abundances of each *nir* gene in 4082 environmental metagenomes across diverse aquatic, terrestrial, host-associated, and engineered biomes. We demonstrate that Nir phylogenies recapitulate ecology distinctly from the corresponding organismal phylogeny. While some clades of the nitrite reductase were equally prevalent across biomes, others had more restricted ranges. Nitrifiers make up a sizeable proportion of the nitrite-reducing community, especially for NirK in marine waters and dry soils. Furthermore, the two reductases showed distinct associations with genes involved in oxidizing and reducing other compounds, indicating that the NirS and NirK activities may be linked to different elemental cycles. Accordingly, the relative abundance and diversity of NirS versus NirK vary between biomes. Our results show the divergent ecological roles NirK and NirS-encoding organisms may play in the environment and provide a phylogenetic framework to distinguish the traits associated with organisms encoding the different lineages of nitrite reductases.

## Introduction

Nitrite is a key intermediate in the global nitrogen (N) cycle, as it is either produced or consumed in most inorganic N transformations. In denitrification, the major route of N loss from the biosphere to the atmosphere, nitrite is reduced to gaseous nitric oxide (NO) by one of two evolutionarily distinct nitrite reductases (hereafter, referred as Nir): the heme-coordinating cytochrome *cd_1_* NirS and the multicopper-oxidase NirK. In addition to structural differences, NirS requires more helper proteins to assemble [1], and it is more likely to co-occur with other genes in the denitrification pathway [2]. Nonetheless, the nitrite-reducing capabilities of NirS and NirK are biochemically redundant in denitrifying organisms, and genes for the two enzymes are rarely found in the same genome [2], although each can compensate for the absence of the other [3, 4]. However, the ratio of genes encoding NirK versus NirS within whole communities varies both between habitats and in response to environmental change [5–13], suggesting divergent ecological roles of the organisms encoding them. This may be in part associated with the diversity of other ecological processes nitrite reductases can be associated with in addition to denitrification, including incomplete denitrification (nitrate/nitric oxide reduction or nitrous oxide reduction [2]); ammonia/hydroxylamine [14, 15], sulphur [16], iron [17], and manganese oxidation [18]; nitric oxide pool replenishment for hydrazine synthase in anaerobic ammonium oxidation (anammox) [19, 20]; nitrite [21, 22] and selenite detoxification [23]; pathogenesis [24]; and magnetite [25, 26] and oxygen generation [27]. These alternative roles may explain the differences in the NirK and NirS communities across biomes [28, 29]. While earlier work suggests a complex evolutionary history for NirS and NirK [30], the massive increase of sequencing data over the past decade offers new possibilities to gain insights into the structure–function relationships of these proteins within an eco-evolutionary context.

**Figure 1:**
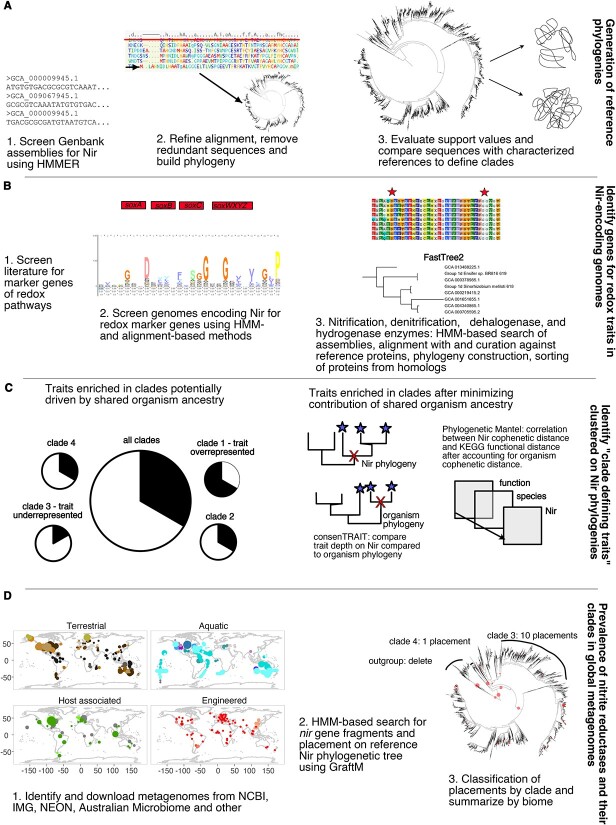
Methods overview diagram. A) generation of reference alignment B) phylogenetic reconstruction C) evaluation of phylogenetic congruence between Nir and organism phylogenies D) screening metagenomes for *nir* gene fragments. HMM logo in panel b was generated in Skylign (http://skylign.org/).

Here, we leveraged publicly available genomes and environmental metagenomes to examine the overall diversity of both NirS and NirK to evaluate the hypothesis that the distribution of Nir types, clades, and subclades across biomes can be explained by environmental conditions (Fig. 1). Specifically, we posit that NirS dominates under conditions more consistently suitable for denitrification, as suggested by both its greater enzyme production cost compared to NirK [10] and its prevalence within organisms capable of completely reducing nitrite to dinitrogen (“complete denitrifiers”; [2]). We further hypothesize that the sequence variation in Nir proteins is linked to ecological differences rather than solely shared organism ancestry [31]. This is primarily rooted in the observation that NirS and particularly NirK phylogenies are incongruent with the 16S rRNA phylogeny [2, 30, 32] and do not reflect the evolutionary history of the organisms harbouring these genes at a finer taxonomic level. To test these hypotheses, we applied a phylogenetic approach to analyse the NirK and NirS sequences obtained by screening >1 000 000 assemblies of isolate and metagenome-assembled genomes, and then used structural features to further categorize NirK and NirS sequence variants into clades. We subsequently identified associations of each Nir clade with the presence of other functional genes found in the genomes of organisms within different clades to infer other terminal electron acceptors and donors that organisms may utilize (i.e. redox traits). This was complemented by a global analysis in which we classified environmental NirS and NirK fragments from 4082 publicly available metagenomes spanning major terrestrial, aquatic, engineered, and host-associated biomes (Table S1) and determined the environmental associations differentiating NirK and NirS as well as the clades comprising each gene. This study provides an unprecedented survey of the diversity and environmental distribution of NO-forming nitrite reductases and shows that the Nir phylogenies recapitulate ecology beyond organismal phylogeny. Such an enhanced understanding of the ecology of nitrite reducers will enable an improved interpretation of future metagenomes regarding this essential function.

## Materials and methods

### Phylogenetic reconstruction of nitric oxide-forming nitrite reductase homologues in genomes

Phylogenies were constructed using a combination of hidden Markov model (HMM)-based approaches and manual curation (Fig. 1A). All bacteria and archaea (7 October 2021), fungi (21 November 2021), and plant and protozoa genome assemblies (19 January 2022) were downloaded from NCBI and were searched for NirK and NirS using “hmmer” [33] against an HMM built from the alignments of the respective protein from Graf *et al.* [2]. Eighteen NirK sequences inferred from foraminifera transcriptomes were also included [34]. After translating the DNA sequences into amino acids and removing identical sequences using CD-HIT (v 4.6; [35, 36]), initial maximum likelihood (ML) trees were reconstructed using FastTree v. 2.1.11 [37].

The resulting Nir trees were then manually pruned to reduce the number of tips while maintaining the breadth of sequence divergence. Sequences were removed if they came from genome assemblies marked as “contaminated” in GenBank, or if they had >5% contamination and/or <90% (80% for eukaryotes) completeness according to BUSCO (v. 5.3.1; [38]. Some lower quality assemblies were retained in poorly sampled regions of the phylogeny. We also excluded NirK sequences identified as occurring on prokaryote-like contigs in eukaryotic assemblies by mmseqs2-taxonomy (v.14; [39]) against the UniRef50 database (release 2021_04; [40]). Taxonomy was assigned to prokaryotic assemblies using GTDB-TK (v. 1.5.0, GTDB R202; [41]), while taxonomy reported with the assembly in NCBI was assigned to eukaryotes. Alignments underwent a final round of manual editing and alignment following the structural information available for NirS [42, 43] and NirK [44] using ARB v. 7.0 [45]. Positions with <5% similarity were excluded from the alignment prior to phylogeny construction, leaving alignments of 573 and 469 amino acids for NirK and NirS, respectively. The best evolutionary model for each protein was determined using modelfinder (LG + F + R10 for NirK and LG + F + R9 for NirS), and phylogenies were subsequently built using IQ-TREE v. 2.1.3 [46]. Node support was determined using the ultrafast bootstrap (*N* = 1000; [46]) and the Shimodaira–Hasegawa approximate likelihood ratio test [47]. Clades were defined with node support values SH-aLRT ≥80% and UFboot ≥95%. In addition to these statistical support criteria, we used protein structure, gene neighbourhood, and previous knowledge on the ecology and/or taxonomy of the encoding organisms to define clades. The clades were named following existing literature where possible [28, 29], reserving Clade 1 of each protein for the “canonical clade” and letters for sub clades within each larger clade. Trees and associated metadata were plotted using iTOL v5 [48].

### Phylogenetic reconstruction of organism history

We built organism phylogenies for bacteria and archaea using a conserved set of 16 ribosomal proteins encoded by single copy genes [49], as facilitated by GToTree v1.7.07 [50]. We excluded eukaryotic sequences from these organism phylogenies and traits analysis due to the lack of reliable protein predictions. Genomes in which fewer than 8 of the 16 targeted ribosomal protein genes were identified were also excluded from the alignment. We subsequently used modelfinder to infer the best amino acid matrix for each partition (Table S2) and identified the most likely tree using IQ-TREE. Due to computing constraints, we manually generated 100 bootstraps of the starting alignment by sampling within each partition and generated an approximate ML tree of each alignment using the WG model in FastTree. Zero-length branches were set to 10× smaller than the smallest edge in the tree to allow consenTRAIT to run. The final phylogeny included 5518 tips for NirK and 528 for NirS.

### Evaluating Nir-niche and Nir-organism phylogenetic congruence

We used a combination of broad metabolic and gene-specific trait approaches to test for ecologically defined clades in the Nir trees. For the broad metabolic characterization, we used EnrichM v.0.6.3 [51] against the KEGG v.10 database to identify orthologues and KEGG pathways that are >90% complete in the assemblies comprising each tree [52] (Fig. 1B). The resulting gene or pathway presence–absence matrix was used to generate Sorensen dissimilarity matrices and was subjected to phylogenetic Mantel tests [53] to evaluate whether the dissimilarity in Nir sequence (i.e. cophenetic distance) correlated with the overall dissimilarity in KEGG orthologue composition while accounting for common organismic history (Fig. 1C). Here, the organism tree was used to constrain the permutations so that more closely related taxa were more likely to switch traits than more distantly related taxa. We used Spearman’s rho as our metric of correlation to account for possible monotonic, nonlinear associations.

Our gene-specific approach focused on the potential to reduce alternative electron acceptors and oxidize inorganic electron donors, reflecting the range of redox states that organisms experience in the environment. We used a combination of HMM searches, phylogenetic reconstruction, and manual inspection of alignments to identify the genes involved in the reduction and oxidation of inorganic compounds (Supplemental Methods). To detect the physiological traits associated with Nir-based nitrite reduction, we searched for traits that were more conserved along the Nir phylogeny than along the organism phylogeny (Fig. 1C). We determined the extent of phylogenetic conservation of the traits using consenTRAIT’s τ_D_ statistic, which is the phylogenetic depth at which 90% of tips share a trait [54]. We calculated an effect size based on Glass’ D [55] for each trait by comparing the observed τ_D_ across 100 bootstrap trees to the τ_D_ expected under the null model where traits are distributed at random on the phylogeny. This allowed us to account for both the uncertainty in the phylogeny structure and undersampled tips as well as compare across phylogenies with different scales. Traits with larger positive effect sizes on the Nir than organism phylogeny were identified as specifically associated with Nir evolution. All redox traits were subsequently subjected to chi-square and *post hoc* chi-square tests to identify those clades that were more commonly associated with a given trait than expected by chance (*α* = 0.05). These were reported as “clade defining traits” (Fig. 1C). All analyses were completed in R v.4.2.0 [56]

### Metagenomic analyses

We searched for publicly available metagenomes sequenced using Illumina platforms that were ≥100 000 reads, ≥150 nucleotides long, and accompanied by sample metadata, focusing on large sequencing projects that had used a standardized metadata collection protocol. The resulting 4082 metagenomes were categorized into four main groups: terrestrial, aquatic (both water and sediments), host-associated (plant and animal, excluding humans), and engineered (Table S1 and Fig. 1D). Terrestrial metagenomes were then assigned to biomes following The Nature Conservancy Terrestrial Ecoregions [57] using the R packages sp v.1.4-5 [58], rgdal v.1.5-23 [59], and rgeos v.0.5-5 [60]. Agricultural soils were excluded from the biome-based approach and were instead categorized as croplands. Marine metagenomes were assigned to biomes based on latitude (polar: latitude >60°; mid-latitude: 30–60°; low-latitude: 0–30°) and freshwater environments were split based on author descriptions of sites. Quantitative metadata were converted into the same units where possible to enable comparison across studies.

### Phylogenetic placement of environmental *nir* reads

To identify the *nirK* and *nirS* fragments in metagenomes, we used GraftM v.0.13.1 [61] which uses an HMM search of translated amino acid sequences followed by placement on a phylogenetic tree to assign reads to clades or taxa (Fig. 1D). A package was built for each protein after calculating the substitution model parameters in “RAxML” v.7.7.2 [62] and rerooting the respective phylogenies in FigTree v.1.4.4 [63]. Comparable outputs from GraftM require that read length is consistent among samples and therefore only the first 150 nucleotides were used. We tested that these parameters were appropriate by completing GraftM on >200 000 150 nt fragments of full-length *nirK* and *nirS* genes and their homologues curated from a large MAG database [64]. Sensitivity was calculated as the fraction of ingroup reads detected by the process, and it was 76% for *nirK* and 93% for *nirS*. Specificity was defined as the fraction of outgroup reads correctly placed in the outgroup, and it was 97% for *nirK* and 100% for *nirS*.

We then used the gappa v.0.8.0 [65] and pplacer v.1.1.19 [66] phylogenetic placement software suites to retain reads where at least 95% of the placement likelihood fell within ingroups (for overall counts) or specific clades (for clade counts) and a likelihood of zero in the outgroup. Placements in the halophilic archaea NirS-like clade, which lacks a cytochrome c domain, were excluded from total *nirS* counts. We then evaluated the relative dominance of *nirK* versus *nirS* in biomes using a method which accounts for both the difference in the relative abundance of these genes and the overall prevalence of *nir* within metagenomes, including those for which *nirK* and/or *nirS* were not detected.


$$\left(\mathrm{\delta} nir={10}^9\ast \left(\frac{ nirK- nirS}{\mathrm{total}\ \mathrm{reads}\ast 150\mathrm{bp}}\right)\right)$$

To evaluate how the Nir diversity varies across habitats, we calculated the balance-weighted phylogenetic diversity (BWPD) using guppy with placements weighted according to their relative abundance [66, 67]. We performed detrended correspondence analysis using vegan (v. 2.6-4; [68]) to visualize biome-level differences in the clade composition for each Nir type using the average of 100 rarefactions at a depth of 15 placements. We also calculated phylogenetic edge correlations [65] for a finer-scale examination of how nitrite reductase carrying communities differ over environmental gradients known to affect nitrite reductase-carrying communities. For soils, we examined the ammonium, nitrate, soil organic carbon (SOC), soil moisture, copper, and iron content, and pH and soil texture (percent sand). For marine water column samples, we evaluated correlations with ammonium, nitrate and nitrite concentrations, dissolved oxygen, chlorophyll, temperature, and sampling depth. Except for δ*nir*, these analyses used metagenomes with >15 placements and for which we had quantitative metadata (*nirS: n* = 224 aquatic, *n* = 1089 soils; *nirK: n* = 588 aquatic, *n* = 1696 soils) because species dissimilarity methods did not converge with fewer placements. Abundance and alpha diversity of *nirK* and *nirS* were compared between biomes using Benjamini–Hochberg FDR-corrected pairwise comparison of ranks following Kruskall–Wallis.

## Results

### Structure of Nir phylogenies

**Figure 2:**
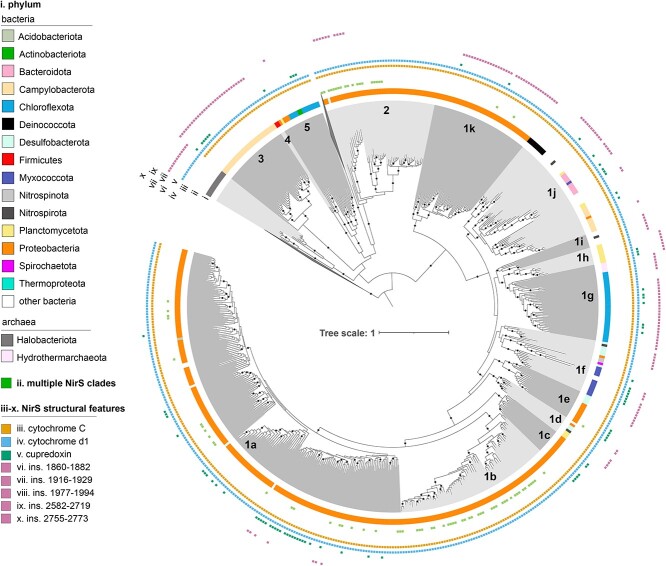
Maximum-likelihood NirS phylogeny. The phylogeny was inferred from 542 full-length NirS amino acid sequences using the LG+F+R9 model in IQ-TREE. Black circles on tree indicate nodes with SH-aLRT ≥ 80% and Ufboot ≥ 95% support. Tips of tree are shaded according to clade, and data rings denote (i) phylum determined using GTDB-TK; (ii) presence of secondary *nirS* copies from other clades; (iii–x) select NirS structural features. The two outgroups, consisting of NirN and NirF sequences, are collapsed for clarity. Clades are numbered and lettered following Wei *et al.* [29] and Bonilla-Rosso *et al.* [28] to the degree possible, starting with the “canonical” Clade 1a [48].

*nirS* was detected in the genomes of 24 bacterial phyla (predominantly Proteobacteria) and Hydrothermarchaeota, with the final phylogeny consisting of 540 NirS sequences (Fig. 2). The two haem d_1_ biosynthesis proteins, NirN and NirF, formed the external and internal outgroups, respectively, to NirS which could be distinguished based on previously described motifs [69] (Fig. S1 and Table S3). A Halobacteriota cytochrome d protein lacking the c-type cytochrome domain of NirS formed an additional outgroup (Supplementary Text). Ingroup sequences fell into 15 clades and could be partially distinguished by the presence of the *nirBEFJNT* genes encoding NirS heme biosynthesis and assembly proteins (Fig. 2 and Table S3). Sixteen percent of the assemblies represented by sequences in the NirS phylogeny encoded multiple copies of NirS, with 85% of these assemblies encoding NirS derived from distinct clades (Table S4).

**Figure 3:**
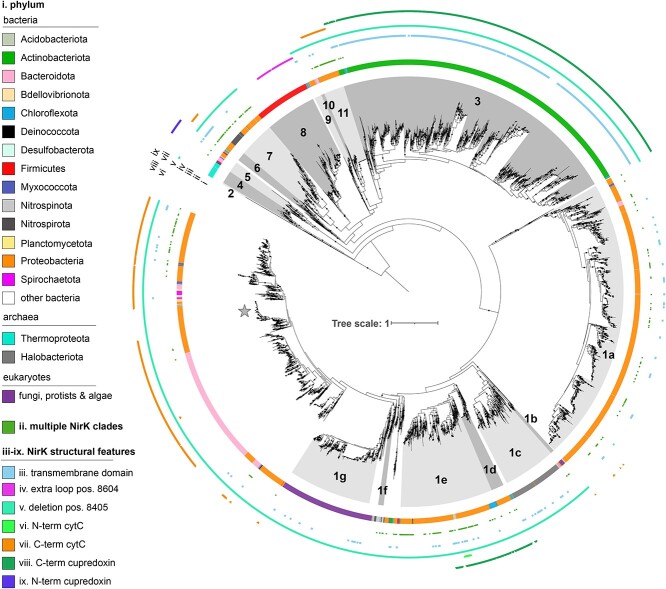
Maximum-likelihood NirK phylogeny. The phylogeny was inferred from 6,422 full-length NirK amino acid sequences using the LG+F+R10 model in IQ-TREE. Black circles on tree indicate nodes with SH-aLRT ≥ 80% and UFboot ≥ 95% support. Tips of tree are shaded according to clade, and data rings denote (i) phylum determined using GTDB-TK; (ii) presence of secondary *nirK* copies from other clades; (iii–ix) select NirK structural features. The outgroup, consisting of 367 multicopper oxidases derived from Cyanobacteria and Thermoprotetota genomes, is collapsed for clarity; clades are numbered and lettered following Wei *et al.* [29] and Bonilla-Rosso *et al.* [28] as much as possible, starting with the “canonical” Clade 1a; the star marks the location of the AniA NirK proteins from *N. gonorrhoeae.* [48]

The NirK phylogeny (Fig. 3) contained much more structurally and taxonomically diverse sequences than the NirS phylogeny, with 6422 NirK sequences derived from all three domains of life, including protists and fungi within eukaryotes, Halobacteriota and Thermoproteota within archaea, and 30 bacterial phyla. The outgroup consisted of 367 multicopper oxidase sequences derived from Cyanobacteria and Thermoproteota and could be distinguished from NirK based on previously identified motifs within the catalytic sites (Fig. S2 and Table S5; [44]). Ingroup sequences fell into 17 clades encompassing 75% of the sequences, with 90% of the remaining sequences occurring in the large, poorly supported region of the phylogeny, including AniA-like NirK sequences. Ten percent of the assemblies represented by sequences in the NirK phylogeny encoded multiple copies of NirK, with 44% of these derived from different clades (Table S6). Co-occurrence of both *nirK* and *nirS* genes was observed in 5.3% of all genome assemblies represented in either tree (Tables S4 and S6).

### Environmental distribution and functional potential of NirS clades

**Figure 4:**
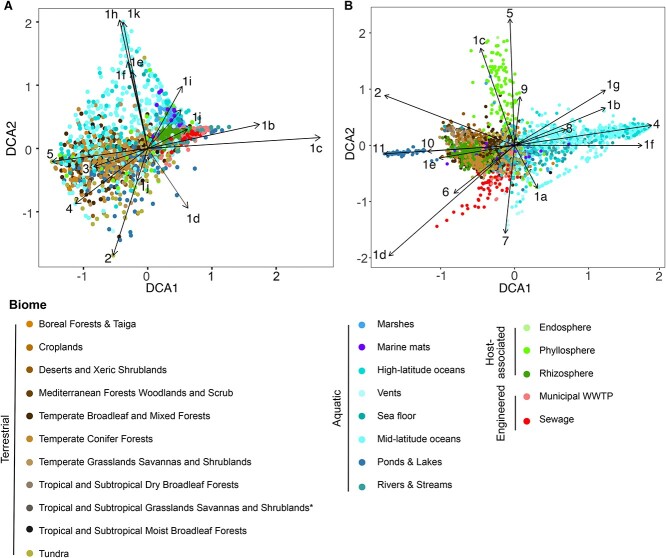
Nitrite reductase composition of globally distributed metagenomes. Ordinations of A) *nirS* and B) *nirK* clade composition based on detrended correspondence analysis. Each point represents the average of 100 rarefactions of 15 placements for a sample. Arrows terminate at the species score for each clade. Biomes represented by fewer than 10 samples after rarefying to 15 placements are excluded from the ordination.

**Figure 5:**
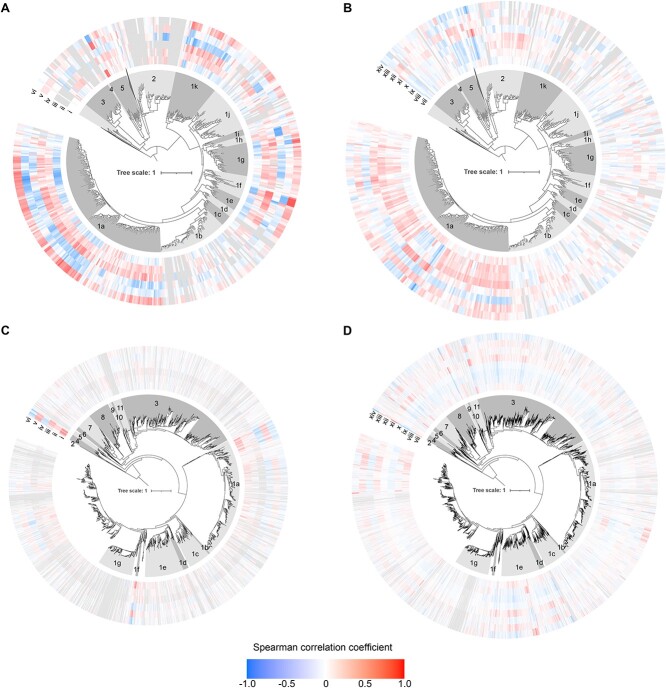
Environmental correlations of nitrite reductases and their clades. Spearman correlation of edge masses on A, B) NirS and C, D NirK phylogenies against environmental variables in A, C) water column seawater and B, D) terrestrial biomes. Values represent correlations in leaves of tree. Outer rings are coloured according to correlation strength, with grey indicating insufficient data. (i) water nitrate + nitrite, (ii) water ammonium, (iii) chlorophyll, (iv) dissolved oxygen, (v) water depth, (vi) water temperature, (vii) soil nitrate, (viii) soil ammonium, (ix) percent SOC, (x) soil moisture, (xi) pH in CaCl2, (xii) sand content, (xiii) copper, and (xiv) iron. These variables were selected for having data available from many metagenomes and were potentially relevant for structuring microbial communities (reactive N availability (i, ii, vii, viii), C availability or input (iii, ix), oxygenation degree (iii, iv, x), and physiochemical properties (v-vi, xi-xiv). Bootstrap support values have been removed for visual clarity. Metagenomes with >15 placements for the gene were included. Since not all metadata are available for all samples, correlations correspond to different subsets of samples [48].

**Figure 6:**
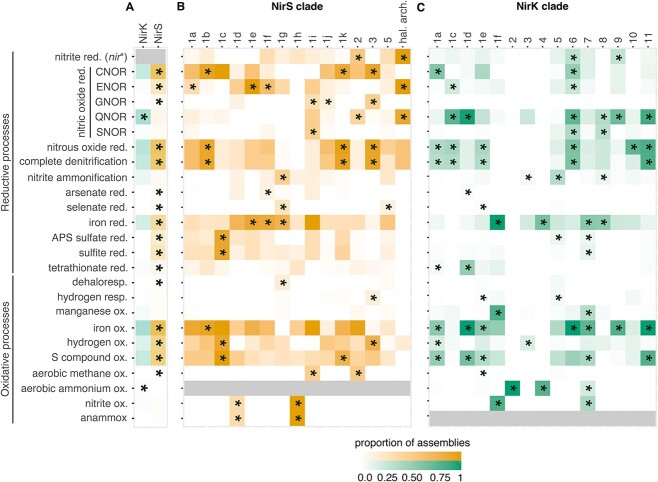
Proportion of assemblies carrying genes for redox traits. A) Proportion for all assemblies carrying the gene for NirS or NirK, or the proportion of assemblies within each clade carrying the genes for the associated function in the B) NirS and C) NirK phylogenies. Shading indicates the fraction of assemblies represented in the reference phylogeny which have the gene or trait of interest. Asterisks denote traits with a greater than expected frequency within a clade based on chi-squared post hoc tests. Grey indicates the trait was not tested due to not being found in members of any clade in the corresponding phylogeny. NirK clades 1b and 1g are not shown because they consist exclusively of eukaryotes and lack reliable protein predictions, while NirS clade 4 is excluded because it consists of just two taxa. nir^*^ refers to *nirS* when discussing assemblies carrying NirK and *nirK* when discussing assemblies carrying NirS. Assemblies carrying both *nirK* and *nirS* genes were excluded from panel A and are shown in Table S8. Genes used as markers for each trait are listed in the Supplementary Methods.

The functional potential and environmental distribution of *nirS* sequences were nonrandomly distributed on its phylogeny. Overall functional dissimilarity based on KEGG orthologues and KEGG pathways correlated with the NirS sequence dissimilarity (phylogenetic Mantel test *r* = 0.21 and *r* = 0.26, respectively; *P* < 0.001). The genomic potential to oxidize or reduce specific compounds (“redox traits”) was likewise heterogeneously distributed on the NirS phylogeny, with hydrogen and iron oxidation, complete denitrification, and the quinol-dependent nitric oxide reduction being more clustered than expected, given organism ancestry (consenTRAIT analysis; Table S7). Biome-level differences in the NirS community composition were similarly apparent (Fig. 4A).Specific NirS clades thus appeared to be functionally distinct and were often associated with specific habitats. The well-characterized proteobacterial Clade 1a made up the majority of reads in many biomes, particularly in engineered biomes but also in croplands and the endosphere and rhizosphere (Fig. S3 and Table S8). This clade shows an overall greater relative abundance under moist, organic matter- or ammonia-rich and low sand content soils, and in marine waters that were warmer, had lower dissolved oxygen, or had higher ammonium and chlorophyll content though not without exception (Fig. 5A and B). Its sister Clade 1b, also predominantly consisting of proteobacterial sequences, showed much weaker correlations with the environmental variables assessed, largely because it was not prevalent in soils or ocean waters (Fig. S4). Rather, this clade was most prevalent in geysers (median 44%) and engineered biomes (Fig. S3) and was characterized by a greater-than-expected association with the potential for iron oxidation and complete denitrification (Fig. 6B and Table S9). Clades 1c (Thiobacillaceae) and 3 (Campylobacterales) consisted of sequences derived from sulphur- and hydrogen-oxidizing lithotrophs, but with different biome preferences; the former was abundant in geysers (24% median relative abundance), while the latter was most abundant in ponds and lakes (15% median relative abundance). Clades derived from sulphur-oxidizing lithotrophs were dominated by marine proteobacterial Clade 1k in the sea floor (8%) and marshes (6%). Clade 1f consisted of sequences derived from Myxococcota among other phyla, and it comprised 3%–11% of *nirS* sequences in saltwater habitats (Fig. S3). Clade 1j consisted of sequences derived mostly from thermophilic bacteria within Deinococcota, Bacteroidota, Campylobacterota, Aquificota, and others (Fig. 2), and it was most prevalent in sewage (4%) and vents (5%; Fig. S3). Clade 1h consisted solely of anammox assemblies from a combination of environments. Although this clade was present at a median abundance of <1% in all biomes, it made up to 39% of reads in seafloor metagenomes, 25% in low latitude oceans, and 20% in rivers. It was also among the more negatively correlated groups with higher oxygen concentrations in ocean waters (Fig. 5A). Clade 2, which consisted of sequences derived from methane-oxidizing Proteobacteria, was often found in organisms with multiple copies of *nirS* and/or both *nirK* and *nirS* (Fig. 2 and Table S4). It accounted for a small but visible proportion of reads and surpassed that of the other methane-oxidizing clade, Clade 1i, which was dominated by sequences derived from Methylomirabilota. Clades 1d, 1e, and 4 were taxonomically diverse but were not prevalent in any biome (Fig. 2 and Fig. S3). Consistent with previous observations, Chloroflexi sequences were polyphyletic [70]. The recently described Chloroflexi *nirS* gene fusion with a cytochrome c551/552 and cytochrome C mono/diheme variant [70] fell into Clade 1g and was rarely observed in the metagenomes. On the other hand, Clade 5, which consisted of sequences derived from a mixture of Chloroflexi, Proteobacteria, Actinobacteriota, and Planctomycetota MAGs and isolates from wastewater and thermophilic environments, dominated all terrestrial biomes except for croplands (22%–38% of sequences; Fig. S3).

### Environmental distribution and functional potential of NirK clades

Similar to NirS, phylogenetic Mantel tests indicated that the overall functional dissimilarity based on KEGG orthologues and pathways correlated with the NirK sequence dissimilarity (*r* = 0.388 and *r* = 0.359, respectively; *P* < 0.001). ConsenTRAIT analysis found that hydrogen, methane, and manganese oxidation, arsenate reduction, and quinol-dependent nitric oxide reduction clustered on the NirK phylogeny (Table S7). Broad differences in *nirK* community composition, particularly between terrestrial and plant versus aquatic environments, were similarly apparent (Fig. 4B).

Specific NirK clades were often characterized by distinct functional genes and were associated with specific environments (Tables S10 and S11; Fig. 5C and D; Fig. S4). The “canonical” proteobacteria-dominated Clade 1a represented a variable proportion of the *nirK* sequences in all biomes (Fig. S4), although it was particularly abundant in drinking water treatment facilities. Subclades of Clade 1a were found at higher relative abundance in nitrate-rich and oxygen-poor ocean waters (Fig. 5C). The remainder of the NirK phylogeny consisted of clades representing all three domains of life, with strong biome-specific associations for some and a more cosmopolitan distribution for others. Among the clades with strong biome-specific associations were Clades 1c, 1d, 5, and 11. Burkholderia Clade 11 was particularly prevalent in ponds and lakes (59% median abundance), and it was strongly associated with genes for nitrous oxide reduction (i.e. *nosZ*) and complete denitrification compared to other clades (Fig. 6C). Clade 1d housed the second genomic copy of *nirK* in Betaproteobacteria genomes and accounted for up to 15% of sequences in sewage. However, this clade rarely occurred in genomes encoding *nosZ* (Fig. 6C and Fig. S4). Clade 5 consisted of sequences derived from a mixture of taxonomically diverse alkaliphilic and thermophilic bacterial genera, including *Thioalkalivibrio, Caldalkalibacillus*, and *Thermus*, the genomes of which frequently encoded the ability for dissimilatory nitrate reduction to ammonium via the formate-dependent nitrite reductase. Consistent with the tolerance to extremophilic conditions known for these organisms, this clade was prevalent in hot springs metagenomes and was also common in the phyllosphere (Fig. S4). Clade 1c was dominated by halophilic archaea with small subclades of Proteobacteria and Bacteroidota (Fig. 3), and it was prevalent in the phyllosphere and in marshes (Fig. S4). Organisms within Clade 1c were often complete denitrifiers via quinol- or cytochrome c-dependent nitric oxide reductases [71] and the halophile-type nitrous oxide reductase (Fig. 6C; Supplementary Data 3; [72, 73]).

Clades 1e and 3 could be considered as “generalist” clades. Clade 1e consisted of sequences related to the hexameric *Hyphomicrobium denitrificans* and *Bradyrhizobium sp.* ORS 375 NirK [74, 75], and it was abundant across all terrestrial biomes (Fig. S4). This clade also contained the highest concentration of Chloroflexi sequences on the tree, driving the overall high abundance of this clade in wastewater treatment plants (Fig. S4). The large Clade 3 consisted of most sequences derived from Actinobacteriota, and it was prevalent across all biomes except for geysers (Fig. S4). Organisms carrying Clade 3 rarely encoded the nitrous oxide reductase NosZ*,* although a small fraction might perform dissimilatory nitrate reduction to ammonium (Fig. 6B). The prevalence of this clade was generally positively correlated with soil pH or copper content, yet negatively correlated with soil organic matter concentration (Fig. 5D).

**Figure 7:**
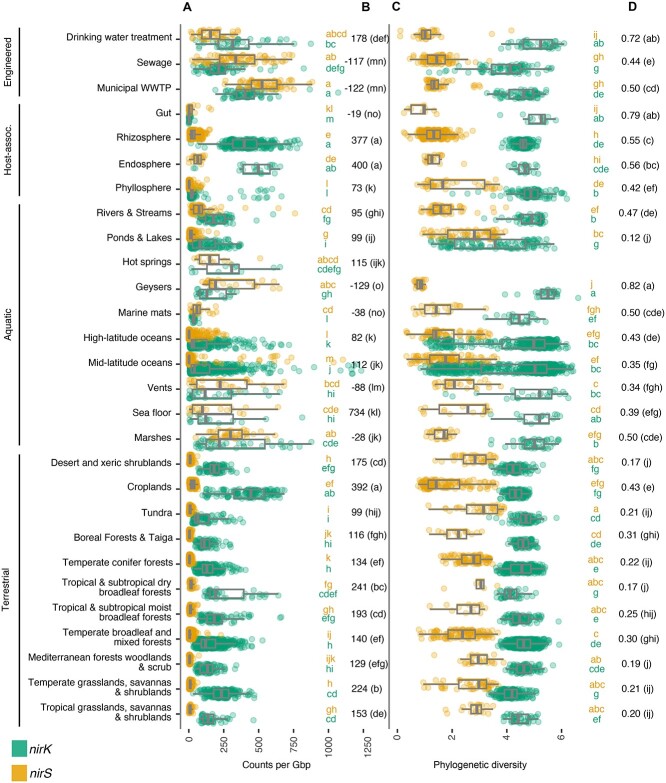
Abundance and diversity of *nir* genes in metagenomes across biomes. A) Abundance of *nirK* and *nirS* genes per gigabase (Gb) sequenced, with B) median difference in *nirK* and *nirS* abundance (δ*nir*). C) Diversity of *nirK* and *nirS* genes based on balance-weighted phylogenetic diversity (BWPD), with D) median ratios of *nirK:nirS* diversity. Ecosystem classifications are shown to the left. Biomes were compared using Benjamini-Hochberg FDR-corrected pairwise ranked comparisons following Kruskall-Wallis. Shared letters denoting similar abundance (in A) or diversity (in C) between biomes are shaded by *nir* type. Biomes represented by fewer than 10 metagenomes were excluded from the figure. Metagenomes off the scale of the axis are not shown but included in calculations for boxplot and pairwise comparisons. Boxplots show median and quartiles, and whiskers show 95 percentiles, and values for individual samples are shown as points.

Nitrifiers fell into six clades in the NirK phylogeny (Figs 3, 7 and Table S6). Clade 1f consisted exclusively of sequences derived from the bacterial nitrite oxidizers and showed strong positive correlations with nitrate and nitrite availability and negative correlations with dissolved oxygen content (Fig. 5C). Clade 7 included all the complete ammonia oxidizers (comammox) and non-nitrifying Proteobacteria, with a handful of ammonia oxidizers. While Clades 7 and 4 dominated various aquatic biomes, Clade 2 was particularly abundant in dry terrestrial biomes and in dry, low SOM, high pH soils (Fig. 5C, D and Fig. S4). This propensity for Clade 2 to be found in dry soils was interesting, given that Nitrososphaera [76] ammonia oxidizing archaea have been reported to be more sensitive to drought than bacterial ammonia oxidizers [77, 78]*.* By contrast, NirK sequences from anammox bacteria were distributed in various regions of the phylogeny outside of these clades (Fig. 3).

Eukaryotic sequences were polyphyletic in the NirK tree [34, 79], with sequences from anoxic sediment- and oxygen minimum zone-dwelling foraminifera forming Clade 1b and with sequences from fungal denitrifiers clustering in Clade 1g (Fig. 3). The remaining eukaryotic sequences were derived from a combination of protists and algae, such as *Naegleria fowleri*, *Spumella vulgaris*, *Galderia sulphuraria*, and *Emiliania huxleyi*, and clustered with Clade 1g. While Clade 1g comprised a median of just 0.1% of the soil *nirK* reads and was not detected in 47% of metagenomes, it accounted for up to 26% of the sequences in ocean waters (median 0.8%; Fig. S4). By contrast, Clade 1b was uniformly rare (Fig. S4).

Most sequences which fell outside our defined clades (ca. 25%) occurred within a taxonomically and structurally diverse group represented in part by the *Neisseria gonorrhoea* AniA (Fig. 3)*.* This group also included sequences from Bacteroidota, many of which are complete denitrifiers and were dominant in sewage and geyser metagenomes, but they were present at low abundance in ocean surface water samples, accounting for a median of 57% and 41% of *nirK* reads in the former and 1% in the latter. Among soils, it was most abundant in tundra (15%) and croplands (12%).

### Comparison of NirS and NirK ecology

**Figure 8:**
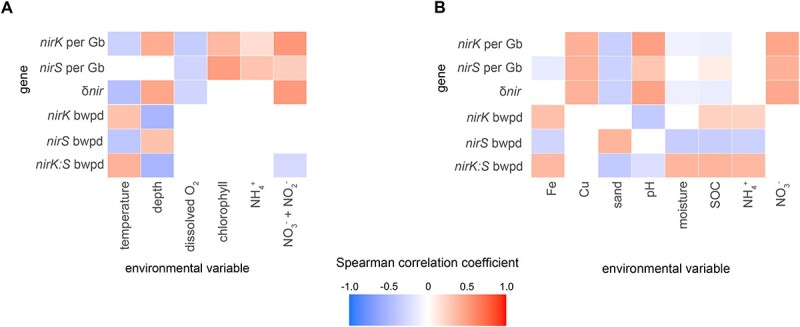
Environmental correlations of prevalence and diversity of nitrite reductase genes. Correlations between environmental variables and the abundance of *nirK* per Gb sequenced, *nirS* per Gb sequenced, ratio of their genes, alpha diversity (bwpd, balance-weighted phylogenetic diversity) and ratio of diversity, in A aquatic and B terrestrial metagenomes. SOC: soil organic carbon.

Based on the redox traits associated with genomes encoding NirS or NirK as well as the biome distribution of *nirS* and *nirK* genes, potential differences in the ecology of organisms became apparent (Figs 6–8). Genomes carrying only *nirS* compared to those with only *nirK* were generally more likely to have all redox traits considered, except for aerobic ammonium oxidation and the quinol-dependent nitric oxide reductase (Fig. 6A). Cross-biome differences in the overall relative abundance of the two nitrite reductases showed that both *nir* genes were highly prevalent in engineered habitats and were rare on leaves and in the animal gut (Fig. 7A). However, *nirK* and *nirS* counts showed divergent biome associations, with *nirK* being more abundant than *nirS* in most biomes. Indeed, median *nirK* abundance was 19 times greater than that of *nirS* per Gb sequenced across all terrestrial biomes, and it was particularly dominant in croplands and the rhizosphere. By contrast, *nirS* was only slightly more abundant than *nirK* in engineered biomes, vents, sea floor, geysers, hot springs, and marshes and dominated most over *nirK* in geysers and wastewater (Fig. 7B). In pelagic marine samples, *nirS* was 34 times less prevalent than *nirK*. The levels of nitrate or nitrite were positively correlated with *nirK* and *nirS* counts and the prevalence of *nirK* over *nirS* (δ*nir*) in both soils and marine samples (Fig. 8; Tables S12 and S13). Variables indicative of environment redox state, like dissolved oxygen in marine samples and soil moisture in soil samples, were negatively correlated with the abundance of both nitrite reductases, and soil moisture indicated the prevalence of *nirS* over *nirK* (negative δ*nir*) at lower redox potentials. Yet, genome comparison indicated that organisms carrying both *nir* genes may be better adapted to low redox conditions than those encoding just one or the other, considering their genomes are also more likely to encode the potential for most redox traits examined (Table S14).

BWPD [67] was also biome-specific for the two *nir* genes (Fig. 7C and D). Metagenomes from drinking water treatment and geysers were characterized by high *nirK* but low *nirS* diversity resulting in a high *nirK:nirS* diversity ratio, while ponds were characterized by among the highest *nirS* but lowest *nirK* diversity and thus a low *nirK:nirS* diversity ratio. Abundance and diversity were negatively correlated for both *nirK* and *nirS* (*r* = −0.45 and −0.57, respectively; *P* < 0.001), indicating that the environments where nitrite reduction capacity is most beneficial particularly benefits only a restricted part of the nitrite reductase phylogeny. For NirS, the decrease in diversity could largely be attributed to an enrichment of Clade 1k in aquatic systems, while for NirK, it depended on biome. Diversities of *nirS* and *nirK* tended to show contrasting correlations with the abiotic variables examined, including an increase in the *nirK:nirS* diversity ratio in soils characterized by high iron, SOC, and/or ammonium contents (Fig. 8).

## Discussion

This study provides novel insights into the phylogenetic structure of two NO-forming nitrite reductases and their distribution across global biomes. We demonstrate that NirK and NirS are diverse proteins whose evolution is associated with both ecological niche (Figs 4 and 6) and organismal phylogeny (Figs 2 and 3), and that genes encoding these two nitrite reductases have strong biome associations (Figs 5, 7, 8 and Figs S3, S4). We further show that despite performing the same chemical reaction, organisms carrying multiple copies, clades, or even classes of nitrite reductases are not rare (Tables S4 and S6). Our results contest the original interpretation of the two kinds of nitrite reductase as functionally redundant [1]. Not only did we find that it is 5–10 times more common than previously thought for genomes to encode multiple nitrite reductases [2], but genes for the two variants also show divergent biome prevalence and correlations with environmental variables (Figs 7 and 8). Usually thought as exclusively associated with anaerobic respiration, nitrite reduction can also be involved in nitrite detoxification [22, 80] or simply support the elimination of excess reducing power to allow other physiological processes to continue [81]. Although the physiological benefits of producing multiple nitrite reductases is an area of active research [4, 21, 82], in principle, it could allow organisms to continue to denitrify across a wider range of environments or under rapidly shifting conditions. The observation that assemblies carrying both *nirS* and *nirK* were more likely to encode genes for many of the other traits examined supports the hypothesis that organisms carrying both have a greater degree of redox flexibility than assemblies carrying *nirK* or *nirS* alone, which should be addressed in future research.

Adaptation to shifting environmental conditions is evident in the conditions under which the two genes and their constituent clades were most prevalent. Biomes such as sewage, WWTP, and marshes were characterized by a high overall nitrite reductase abundance and were often dominated by *nirS*, and particularly the “canonical” Clade 1a, while soils and pelagic aquatic biomes tended to be dominated by *nirK*. Previous studies on the ecology of nitrite reductases often concluded that *nirS* abundance and/or diversity are more sensitive to changing environmental conditions than *nirK* [11, 13, 24, 83, 84]. This contrasts with our results showing similar or lower correlations between *nirS* abundance or diversity and environmental factors. Our results also contrast the observations that *nirK* diversity increases with soil pH [11] and *nirS* diversity increases with ammonium content [9]. These previous studies are largely primer-based and constrained to evaluating the response of Clade 1a of both genes, which our work demonstrates is particularly poorly representative of the *nirK*-type nitrite reductase community. However, consistent with our observations, a metagenome-based study found that wet, anoxic soils favour *nirS* over *nirK*, which the authors attributed to the greater biosynthetic cost of the former [10]. The positive correlation with moisture was driven by a decrease in *nirK* prevalence rather than increased *nirS* in the present study, and it largely corresponded to a reduction in the diversity of *nirS* and a positive correlation with moisture for *nirS* Clade 1a. We propose that the reduction in *nirK* counts in moist soils may also be driven by the oxygen requirement of organisms with this Nir variant, including the ammonia-oxidizing archaea encoding NirK clade 2 as well as groups of Actinobacteriota or fungi which reduce nitrite at a similar or more rapid rate in the presence of oxygen [85, 86]. It is also possible that mechanisms driving the relative prevalence of *nirK* and *nirS* differ between the intercontinental scale of our analysis and the more localized scales of earlier studies.

By expanding the phylogeny to include diverse eukaryotic NirK sequences and extending the study into additional biomes, we confirmed that, contrary to primer-based approaches that also pick up a substantial number of bacterial *nirK* reads [87–89], fungal nitrite reductase sequences are rare in soil [90]. However, our study found them to be occasionally abundant in the marine water column. Given that known fungal denitrifiers have an oxygen requirement, the sporadic prevalence within pelagic samples may be attributed to the overrepresentation of samples derived from well-oxygenated surface waters within our database (90% of samples have >4 mg O_2_/l). Overall, our analysis provides a global assessment and adds nuance to some of the previously reported drivers of NirK and NirS diversity and abundance, which were restricted to specific habitats or clades of the nitrite reductase phylogenies not necessarily representative of the full diversity of organisms carrying each gene.

Finally, our study addressed the co-occurrence of NirS and NirK subclades with other functional genes, such as those involved in iron and sulphur redox reactions. In this analysis, we did not apply characteristic motif verification [91] as we did for the Nir sequences, so the presence of these additional genes may be overestimated. Nonetheless, our analysis shows that organisms encoding NirS, as opposed to NirK, display the genetic potential for reducing and oxidizing a broad range of substrates, although this varies between clades. Of note is the potential for the further processing of nitric oxide into the greenhouse gas nitrous oxide by nitric oxide reductase and eventually to N_2_ by NosZ. Previously, the cytochrome c-dependent nitric oxide reductase was proposed to be exclusively involved in denitrification, while the quinol-dependent variant may also detoxify nitric oxide [92]. Accordingly, assemblies carrying NirS more commonly encode the cytochrome c-dependent nitric oxide reductase and NosZ (i.e. potential for complete denitrification), and there is a much lower prevalence of *nosZ* among assemblies carrying *nirK*. This highlights the divergent roles that NirK and NirS may play in the environment. Yet, this broader pattern of reduced potential for complete denitrification among NirK microorganisms was largely driven by the absence or near absence of the complete denitrification trait among NirK Clades 2–4, represented by ammonia-oxidizing archaea and Actinobacteriota. This includes the absence of Nor in many cases, indicating that these organisms must either tolerate or process the potentially toxic NO in another way [27, 93]. The weak co-association between NirK and the cytochrome c-dependent nitric oxide reductase, as well as NosZ, combined with the much higher abundance of *nirK* than *nirS* across most biomes further indicate that Nir-driven NO-forming nitrite reduction is not solely associated with “canonical” denitrification, particularly in marine surface waters and dry soils. For example, it has been suggested that NirK in the bacterial ammonia oxidizer *Nitrosomonas europaea* may catalyze the oxidation of nitric oxide to nitrite [94]. This suggests a cautious interpretation of denitrification capacity in count-based analyses of environmental metagenomes. Our work provides a framework beyond gene annotation that integrates phylogenetic background, protein structure, genomic context, and environmental distributions for analysing environmental *nir* genes based on clade-level information that can help infer the fate of nitrite and the processes to which its reduction is most likely to be linked to. Nitrite reduction is a complex ecological trait, and our research implies its understanding and effective management necessitates considering it as such.

## Supplementary Material

Supplementary_Figure_1_ycae020

Supplementary_Figure_2_ycae020

Supplementary_Figure_3_ycae020

Supplementary_Figure_4_ycae020

Supplementary_File_1_ycae020

Supplementary_File_2_ycae020

Supplementary_Materials_and_Methods_ycae020

## Data Availability

All genomes and metagenomes used in this analysis are publicly available and documented with their sources in Tables S1, S4, and S6. Reference alignments, clade-specific HMMs, and phylogenies for each protein are available in figshare (doi:10.6084/m9.figshare.23913078).
