# Supplementary material for: Phylogenetics and environmental distribution of nitric oxide-forming nitrite reductases reveal their distinct functional and ecological roles
Source: ISME Commun. 2024 Feb 2;4(1):ycae020. doi: 10.1093/ismeco/ycae020 (PMC10999283; doi:10.1093/ismeco/ycae020)
Supplement: Supplementary_Figure_1_ycae020 [file supplementary_figure_1_ycae020.pdf]

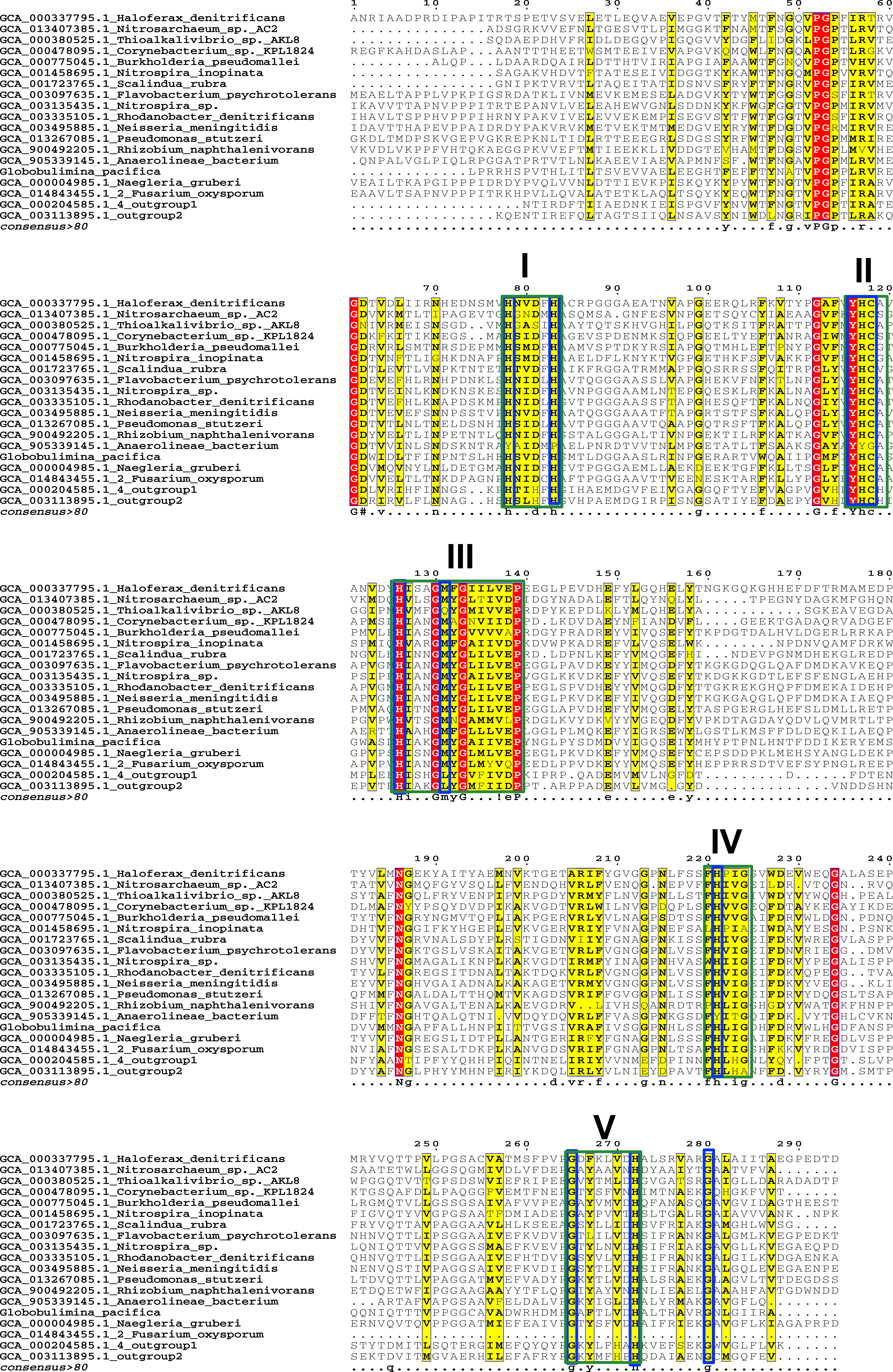

1 10 20 30 40 50 60
GCA_000337795.1_Haloferax_denitrificans ANRIAADPRDIPAPITRTSPETVSVELETLEQVAEVEPGVTFTYMTFNGQVPGPFIRTRV
GCA_013407385.1_Nitrosarchaeum_sp._AC2 ...............ADSGRKVVEFNLTGESVTLPIMGGKTFNAMTFSGQVPGPTLRVTQ
GCA_000380525.1_Thioalkalivibrio_sp._AKL8 ...............SQDAEPDHVFRIDLDEGQVPIGQGVVYDGFLIDGKLPGPTLRVTE
GCA_000478095.1_Corynebacterium_sp._KPL1824 REGFKAHDASLAP...AANTTTHEETWSMTEEIVEVAPGVKQMQWLFNGQAPGPTLRGKV
GCA_000775045.1_Burkholderia_pseudomallei ....ALQP...LDAARDQAIRLDTTHTVIRIAPGIAFAAWTFGNQVPGPTVHVKV
GCA_001458695.1_Nitrospira_inopinata ................SAGAKVHDVTFTATESEIVIDGGTKYKAWTFNGQMPGPVVRVTQ
GCA_001723765.1_Scalindua_rubra ................GNIPKTVRVTLTAQEITATIDSNVSFRYFTFNGRVPGPFIRVME
GCA_003097635.1_Flavobacterium_psychrotolerans MEAELTAPPLVPKPIGSRDATKLIVNMEVKEMESELADGVKYTYWTFGGSVPGSFIRTRV
GCA_003135435.1_Nitrospira_sp. IKAVVTTAPNVPPPITRTEPANVLVELEAHEWVGNLSDDNKYKFWGFGGTVPGPMIRVMV
GCA_003335105.1_Rhodanobacter_denitrificans IHAVLTSPPHVPPPIHRNYPAKVIVELEVVEKEMPISEGVSYTFWTFGGTVPGSFIRVRQ
GCA_003495885.1_Neisseria_meningitidis IDAVTTHAPEVPPAIDRDYPAKVRVKMETVEKTMTMEDGVEYRYWTFDGDVPGRMIRVRE
GCA_013267085.1_Pseudomonas_stutzeri GKDLTMDPSKVGEPVGKREPKNLTIDLRTTEEEGRLSDGSSYRFWTFNGTVPGPMMRIRE
GCA_900492205.1_Rhizobium_naphthalenivorans VKVDLVKPPFVHTQKAEGGPKVVEFTMTIEEKKLIVDDGTEVHAMTFDGSVPGPLMVVHE
GCA_905339145.1_Anaerolineae_bacterium .QNPALVGLPIQLRPGGATPRTVTLNLKAEEVIAEVAPMNFSF.WTFNGAVPGPMLRVME
Globobulimina_pacifica .............LPRRHSPVTHLITLTSVEVVAELEEGHTFEFFTYNATVPGPPLRVRE
GCA_000004985.1_Naegleria_gruberi VEAILTKAPGIPPPIDRDYPVQLVVNLDTTIEVKPISKQYKYPYWTFNGTVPGPFIRARV
GCA_014843455.1_2_Fusarium_oxysporum EAAVLTSAPNVPPPITRKHPVLLQVALATETKLAQLTSQYKYEQWTFNGTVPGPFIRARV
GCA_000204585.1_4_outgroup1 ....................NTIRDFTIIAEDNKIEISPGVFYNVWTFNGTVPGPTIRATE
GCA_003113895.1_outgroup2 ................KQENTIREFQLTAGTSIIQLNSAVSYNIWDLNGRIPGPTLRAKQ
consensus>80 ....y....f.g.vPGp..r...
I II
70 80 90 100 110 120
GCA_000337795.1_Haloferax_denitrificans GDTVDLIIRNHEDNSMVHNVDFHACRPGGGAEATNVAPGEERQLRFKVTYPGAFVYHCAV
GCA_013407385.1_Nitrosarchaeum_sp._AC2 GDVVKMTLTIPAGEVTGHGNDMHASQMSA.GNFESVNPGETSQYCYIAEAAGVFKYHCSG
GCA_000380525.1_Thioalkalivibrio_sp._AKL8 GNIVRMEISNSGD..VMHGASIHAAYTQTSKHVGHILPGQTKSITFRATTPGVFNYHCAP
GCA_000478095.1_Corynebacterium_sp._KPL1824 GDKFKITIKNEGS..MAHSIDFHAGEVSPDENMKSIQPGEELTYEFTANRAGIWNYHCST
GCA_000775045.1_Burkholderia_pseudomallei GDRVRLSMTNRSDEPMMHSMDFHAAMVSPTDKYRSIAPGQTMHLEFTPNYPGVFNYRCGT
GCA_001458695.1_Nitrospira_inopinata GDTVNFTLIGHKDNAFPHSMDFHAAELDFLKNYKTVGPGETHKFSFVAKKPGVFPYHCGA
GCA_001723765.1_Scalindua_rubra GDTLEVTLVNPKTNTETHTVDFHAIKFRGGATRMMAPPGQSRRSSFQITRPGLYVYHCVG
GCA_003097635.1_Flavobacterium_psychrotolerans GDEVEFHLRNHPDNKLSHNIDLHAVTQGGGAASSLVAPGHEKVFNFKTLNPGLYVYHCAT
GCA_003135435.1_Nitrospira_sp. GDTVEINLKNDKNSKESHNIDFHAVNPGGGAAMLNTEPGQESKLRFKALNAGLYPYHCAT
GCA_003335105.1_Rhodanobacter_denitrificans GDTVEFHLKNAPDSKMPHNIDLHGVTPGGGAASSFTAPGHESQFTFKALNQGIYVYHCAT
GCA_003495885.1_Neisseria_meningitidis GDTVEVEFSNNPSSTVPHNVDFHAATQGGGAAATFTAPGRTSTFSFKALQPGLYIYHCAV
GCA_013267085.1_Pseudomonas_stutzeri GDTVTLNLTNELDSNHIHSIDLHAVTPGGGAAVTQAAPGQTRSFTFKALQPGLYVYHCAT
GCA_900492205.1_Rhizobium_naphthalenivorans GDYVELTLINPETNTLQHNIDFHSSTALGGGALTIVNPGEKTILRFKATKAGVFVYHCAP
GCA_905339145.1_Anaerolineae_bacterium GDTVVINLSNDSKNTRAYAIDMPAELPNRDTVVTNLMPGETATLTFSAAKSGAYAYYGAG
Globobulimina_pacifica GDWIDLTFINPNTSLHEHSVDFHSMTLDGGAASLRINPGERARTVWQAIIPGMFIYHCAS
GCA_000004985.1_Naegleria_gruberi GDVMQVNYLNLDETGMAHNIDFHCVTPGGGAEMLLAEKDEEKTGFFKLLTSGLFIYHCAA
GCA_014843455.1_2_Fusarium_oxysporum GDVVELTLTNKDPAGNPHNIDCHAFTPGGGAAVTTVEENESKTARFKLLYPGLYPYHCAA
GCA_000204585.1_4_outgroup1 GDLVRIHFINNGS..KPHTIHFHGIHAEMDGVFEIVGAGGQFTYEFVAGPVGVHPYHCHV
GCA_003113895.1_outgroup2 GDRIRVLFLNNAG..HSHSLHFHGVHPAEMDGIRPISNGSATIYEFDAEPYGVHIYHCHI
consensus>80 G#.v....n.......h.d.h................g.....f.....G.f.Yhc..
III
130 140 150 160 170 180
GCA_000337795.1_Haloferax_denitrificans ANVDYHISAGMFGIILVEPEEGLPEVDHEFYLGQHELYTNGKGQKGHHEFDFTRMAMEDP
GCA_013407385.1_Nitrosarchaeum_sp._AC2 VKMDQHVLSGMYGLTIVDPIDGYNADALEFTLQYNQLYL.....TPEGNYDAGKMFGHQN
GCA_000380525.1_Thioalkalivibrio_sp._AKL8 GGIPNHVMFGQYGMIVVEPRDPYKEPDLKLYMLQHELYA...........SGKEAVEGDA
GCA_000478095.1_Corynebacterium_sp._KPL1824 APMSIHIANGMAGNVIIDPPD.LKDVDAEYNFIANDVFL....GEEKTGADAQRVADGEF
GCA_000775045.1_Burkholderia_pseudomallei PMVLEHIASGMYGVVVVAPRDGYPRADREYVIVQSEFYTKPDGTDALHVLDGERLRRKAP
GCA_001458695.1_Nitrospira_inopinata SPMIQHVARGMFGAIIVDPKDVWPKADREFVLVQSELWK.........NPDNVQAMFDRKW
GCA_001723765.1_Scalindua_rubra NGVLHHINNGMYGLILVEPRD.LDPNLKEFYVMQGEFHI...NDEVPGNMDHEKGLREDP
GCA_003097635.1_Flavobacterium_psychrotolerans APVGNHIANGMYGLILVEPEGGLPAVDKEYYIMQGDFYTKGKGDQGLQAFDMDKAVKEQP
GCA_003135435.1_Nitrospira_sp. PSIPPHIANGMYGAILVEPVGGLKKVDKEFYVVQSEFYTKDGKKGDTLEFSFENGLAEHP
GCA_003335105.1_Rhodanobacter_denitrificans APVGNHIANGMYGLILVEPPEGLSPVDHEYYVMQGDFYTTGKREKGHQPFDMEKAIDEHP
GCA_003495885.1_Neisseria_meningitidis APVGNHIANGMYGLILVEPKEGLPKVDKEFYIVQGDFYTKGKGAQGLQPFDMDKAIAEQP
GCA_013267085.1_Pseudomonas_stutzeri PMVAQHITNGMYGLILVEPEGGLAPVDHEFYVMQGELYTASPGERGLHEFSLDMLLRETP
GCA_900492205.1_Rhizobium_naphthalenivorans PGVPWHVTSGMNGAMMVLPRDGLKVYDKVYYVGEQDFYVPKDTAGDAYQDVLQVMRTLTP
GCA_905339145.1_Anaerolineae_bacterium AERTTHAAHGMFGLLLVEPLGGLPLMQKEFYIGRSEWYLGSTLKMSFFDLDEQKILAEQP
Globobulimina_pacifica GWASIHIAKGMYGAIIVEPYNGLPYSDMDVYIGQSEIYMHYPTPNLHNTFDDIKERYEMS
GCA_000004985.1_Naegleria_gruberi GPVPSHISNGMYGLMLVEPEEGLPKVDKEFYVMQSEFYCEPSDDPKLMEHSYANGLDEKP
GCA_014843455.1_2_Fusarium_oxysporum APVPVHIANGMYGLMYVQPEEDLPPVDKEYYVMQSEFYHEPPRRSDTVEFSYPNGLREEP
GCA_000204585.1_4_outgroup1 MPLEEHISHGLYGVFIVDPKIPRP.QADEMVMVLNGFDT.........D.....FDTEN
GCA_003113895.1_outgroup2 EPVTPHIAKGLYGMFIIDPPT.ARPPADEMVLVMG.GYD...............VNDDSHN
consensus>80 .....Hi..GmyG...!eP.........e......e.y.....................
IV
190 200 210 220 230 240
GCA_000337795.1_Haloferax_denitrificans TYVLMNGEKYAITYAEMNVKTGETARIFYGVGGPNLFSSFHPIGSVWDEVWEQGALASEP
GCA_013407385.1_Nitrosarchaeum_sp._AC2 TATVVNGMQFGYVSQLLFVENDQHVRLFVENQG.NEPVFFHIVGEILDR.VTQGN..RVQ
GCA_000380525.1_Thioalkalivibrio_sp._AKL8 SYTAFNGQLFRYVENPIPVRPGDYVRMYFLNVGPNLLSTFHIVGIVWDYVYWQGH.PEAL
GCA_000478095.1_Corynebacterium_sp._KPL1824 DLMAFNYYPSQYDVDPIKAKVGDTVRLWILNVGPDQPLSFHVVGEQFDTAYKEGAYIKDE
GCA_000775045.1_Burkholderia_pseudomallei TYTVFNGRYNGMVTQPLIAKPGERVRLYVLNAGPSDTSSFHVVGAIFDRVWLDGN.PDNQ
GCA_001458695.1_Nitrospira_inopinata DHTVFNGGIFKYHGEPLEVKVGERVRIYFVNAGPNEFSALHPIAEIWDAVYESGN.PSNK
GCA_001723765.1_Scalindua_rubra DYVVFNGRVNALSDYPLRSTIGDNVIIYFGNAGPNKISSFHIVGEIFDKVWREGVLASPP
GCA_003097635.1_Flavobacterium_psychrotolerans DYVVFNGKTGSLVSKAITAKVGETVRLFVGNGGPNLVSSFHVIGEIFDNVRIEGG..DMV
GCA_003135435.1_Nitrospira_sp. SHVVFNGMAGALIKNPLKAKVGDTIRMFYINAGPNHVASWHIIGEIFDRVYPEGALISPP
GCA_003335105.1_Rhodanobacter_denitrificans TYVLFNGREGSITDNALTAKTDQKVRLFVGNGGPNLVSSFHVIGEIFDKVQPEGG..TVA
GCA_003495885.1_Neisseria_meningitidis EYVVFNGHVGAIADNALKAKAGETVRMYVGNGGPNLVSSFHVIGEIFDKVYVEGG..KLI
GCA_013267085.1_Pseudomonas_stutzeri QFMMFNGALDALTTHQMTVKAGDSVRIFFGVGGPNLISSFHVIGEIFDRVYDQGSLTSAP
GCA_900492205.1_Rhizobium_naphthalenivorans SHIVFNGAVGALTENALKAEVGDRV..LIVHSQANRDTRPHLIGGHGDYVWATGKFHNPP
GCA_905339145.1_Anaerolineae_bacterium DFFTFNGHTQALQTNI.VVDQYDQVRLFFVASGPNKGSDFYITGQIFDK.VYTGHLCVKN
Globobulimina_pacifica DVVMMNGAPFALLHNPIIITTVGSIVRAFIVSGGPNHLSSFHLIGDHFDRVWLHGDFANSP
GCA_000004985.1_Naegleria_gruberi TYVVFNGREGSLIDTPLLANTGERIRFYFGNAGPNLSSSFHVIGAIFDKVFREGDLVSPP
GCA_014843455.1_2_Fusarium_oxysporum NVIAFNGSESALTDKPLKANVGDSVRIFFGNAGPNLTSAFHIIGSHFKKVYRDGDVISPP
GCA_000204585.1_4_outgroup1 NFYAANTIPFYYQHHPIQINTNELIRIYVVNMEFDPINNFHLHGNLYQY.FPTGT.SLVP
GCA_003113895.1_outgroup2 DYYAFNGLPHYYMHNPIRIYKDQLIRLYVLNIEYDPAVTFHLHANFFD.VYRYGM.SMTP
consensus>80 .....Ng...............d.vr.f....g.n....fh.ig...d.....G......
V
250 260 270 280 290
GCA_000337795.1_Haloferax_denitrificans MRYVQTTPVLPGSACVATMSFPVPGDFKLVDHALSRVARGALAIITAEGPEDTD
GCA_013407385.1_Nitrosarchaeum_sp._AC2 SAATETWLLGGSQGMIVDLVFDEPGAYAAVNHDYAAIYTGAATVFVA.......
GCA_000380525.1_Thioalkalivibrio_sp._AKL8 WPGGQTVTTGPSDSWVIEFRIPEEGVYTMLDHGVGATSRGAIGLLDARADADTP
GCA_000478095.1_Corynebacterium_sp._KPL1824 KTGSQAFDLLPAQGGFVEMTFNEPGTYSFVNHIMTNAEKGQHGKFVVT......
GCA_000775045.1_Burkholderia_pseudomallei LRGMQTVLLGSSGSAIVAFVVPEAGAYVMVDHQFANASQGAVGVIDAGTHEEST
GCA_001458695.1_Nitrospira_inopinata FIGVQTYVVGPGSAATFDMIADEPGAYPVVTHSLTGALRGAIAVVVANK..NPK
GCA_001723765.1_Scalindua_rubra FRYVQTTAVPAGGAAVLHLKSEEAGSYLLIDHSVFRIAKGAMGHLWVSG.....
GCA_003097635.1_Flavobacterium_psychrotolerans NHNVQTTLIPSGGAAIVEFKVDVPGTLILVDHSIFRANKGALGMLKVEGPEDKT
GCA_003135435.1_Nitrospira_sp. LQNIQTTVVPAGGSSMAEFKVEVPGTYLNVDHSIFRIAKGAVGLLKVEGAEQPD
GCA_003335105.1_Rhodanobacter_denitrificans QHNVQTTLIPSGGAAIVEFHTDVPGSYVLVDHSIFRANKGALGILKVDGAENKA
GCA_003495885.1_Neisseria_meningitidis NENVQSTIVPAGGSAIVEFKVDIPGSYTLVDHSIFRANKGALGQLEVEGAENPE
GCA_013267085.1_Pseudomonas_stutzeri LTDVQTTLVPAGGATMVEFVADYPGKYTLVDHALSRAEKGLAGVLTVTDEGDSS
GCA_900492205.1_Rhizobium_naphthalenivorans ETDQETWFIPGGAAGAAYYTFLQPGIYAYVNHNLIEAELGAAAHFAVTGDWNDD
GCA_905339145.1_Anaerolineae_bacterium ...ARTAFVAPGSAAVFELDALVPGKYIGLDHALYRMAKGAVGFLQ........
Globobulimina_pacifica QQNIQTTTVPPGGCAVADWRHDMPGAFTLVDHALTRAVRGNLGIIRA.......
GCA_000004985.1_Naegleria_gruberi ERNVQVTQVPPGGATMIEFEAIVPGTYSFVDHAIFRIEKGAVGFLKIAGAPRPD
GCA_014843455.1_2_Fusarium_oxysporum ....
GCA_000204585.1_4_outgroup1 STYTDMITLSQTERGIMEFQYQYPGKYLFHAHKVEFSEKGWVGLFLVK......
GCA_003113895.1_outgroup2 SEKTDVITMGVAERHILEFAFRYPGKYMFHPHQDAIAENGCMGQFEV.......
consensus>80 ....q.................g.y....h........g..............

Fig. S1 Representative NirS alignment. Yellow boxes represent >70% conserved residues and red boxes represent identical residues. Numbered red boxes mark characteristic conserved motifs (I, III, IV, V, VII and IV distinguish NirS from NirN and NirF) and blue boxes mark functionally important residues, and asterisks beneath alignment mark specific residues where nirS can be differentiated from NirF and NirN. Notably, motif I on the cyt_c domain was generally characterised by -Ca**G**CHg- in NirS, motif VI by -LHD- (vs. -PyD- in NirF and -LDD- in NirN), and motif VII at the cyt_{d1} domain is identified by the universal absence of a proline in the third position in NirN and NirF (i.e. -PHpGpG- vs. PH!PgeG). Clade 1c: *Hydrogenophilales sp.;* clade 3: *Sulfurimonas paralvinellae;* clade 4: *Calidifontibacillus erzurumensis;* clade 1d: *Brocadia sp. WS118;* clade 1e: *c. Magnetaquicoccus inordinatus;* clade 1a: *Stutzerimonas stutzeri;* clade 1f: *Deltaproteobacteria bact. GWA2_43_19;* clade 5: *Levilinea saccharolytica;* clade 1h: *Scalindua brodae;* clade 1j: *Thermus oshimai;* clade 1b: *Thauera linaloolentis;* clade 2: *Methylomicrobium album;* clade 6: candidatus *Methylomirabilis oxyfera;* clade 1g: *Roseiflexus castenholzii;* clade 1k: *Colwellia psychrerythraea;* halophilic archaea NirS-like: *Natrinema pellirubrum.* The Fig. was prepared in ESPscript/ENDscript.
