# Supplementary material for: Phylogenetics and environmental distribution of nitric oxide-forming nitrite reductases reveal their distinct functional and ecological roles
Source: ISME Commun. 2024 Feb 2;4(1):ycae020. doi: 10.1093/ismeco/ycae020 (PMC10999283; doi:10.1093/ismeco/ycae020)
Supplement: Supplementary_Figure_2_ycae020 [file supplementary_figure_2_ycae020.pdf]

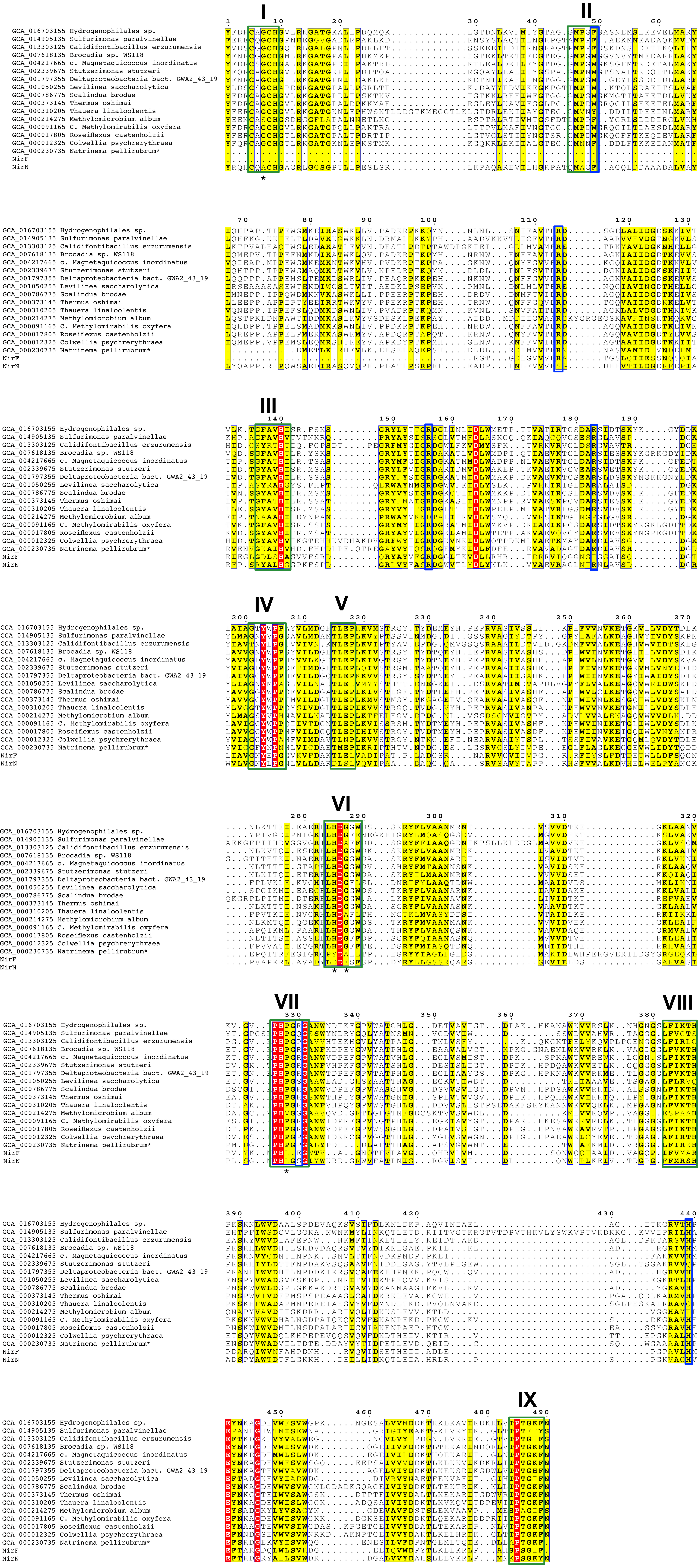

I
II
III
IV
V
VI
VII
VIII
IX
GCA_016703155 Hydrogenophilales sp.
GCA_014905135 Sulfurimonas paralvinellae
GCA_013303125 Calidifontibacillus erzurumensis
GCA_007618135 Brocadia sp. WS118
GCA_004217665 c. Magnetaquicoccus inordinatus
GCA_002339675 Stutzerimonas stutzeri
GCA_001797355 Deltaproteobacteria bact. GWA2_43_19
GCA_001050255 Levilinea saccharolytica
GCA_000786775 Scalindua brodae
GCA_000373145 Thermus oshimai
GCA_000310205 Thauera linaloolentis
GCA_000214275 Methylomicrobium album
GCA_000091165 C. Methylomirabilis oxyfera
GCA_000017805 Roseiflexus castenholzii
GCA_000012325 Colwellia psychrerythraea
GCA_000230735 Natrinema pellirubrum*
NirF
NirN

Fig. S2 Representative NirK alignment. Yellow boxes represent >70% conserved residues and red boxes represent identical residues. Green boxes mark characteristic conserved motifs differentiating NirK from other MCOs (**H**niDf**H**- (or -**H**ni**S**f**H**- in clade 5; [91]) rather than **H**ti**H**F**H**- in motif I; Y**H**Cap- instead of -Y**H**C**H**vm- in motif II; -trp**H**vIG- instead of -insf**H**l**H**G- in motif IV; and -GiYAyd**H**- instead of GkYmF**H**A**H**- in motif V), and blue boxes mark functionally important residues. From top to bottom, the sequences represent: clade 1c: *Haloferax denitrificans*; clade 4: *Nitrosoarcheum sp.;* clade 5: *Thioalkalivibrio thiocyanoxidans*; clade 4: *Nitrososphaera viennensis*; Clade 7: *Nitrospira nitrificans*; clade 1g: *Fusarium oxysporum;* cladeless anammox: *Scalindua rubra;* clade 8: *Paenibacillus amylolyticus;* clade 3: *Propionibacterium australiense*; clade 11: *Burkholderia mallei;* clade 1a: *Nitrosomonas oligotropha*; clade 9: *Rhodanobacter glycinis*. The Fig. was prepared in ESPscript/ENDscript.
