# Supplementary material for: Phylogenetics and environmental distribution of nitric oxide-forming nitrite reductases reveal their distinct functional and ecological roles
Source: ISME Commun. 2024 Feb 2;4(1):ycae020. doi: 10.1093/ismeco/ycae020 (PMC10999283; doi:10.1093/ismeco/ycae020)
Supplement: Supplementary_Figure_4_ycae020 [file supplementary_figure_4_ycae020.pdf]

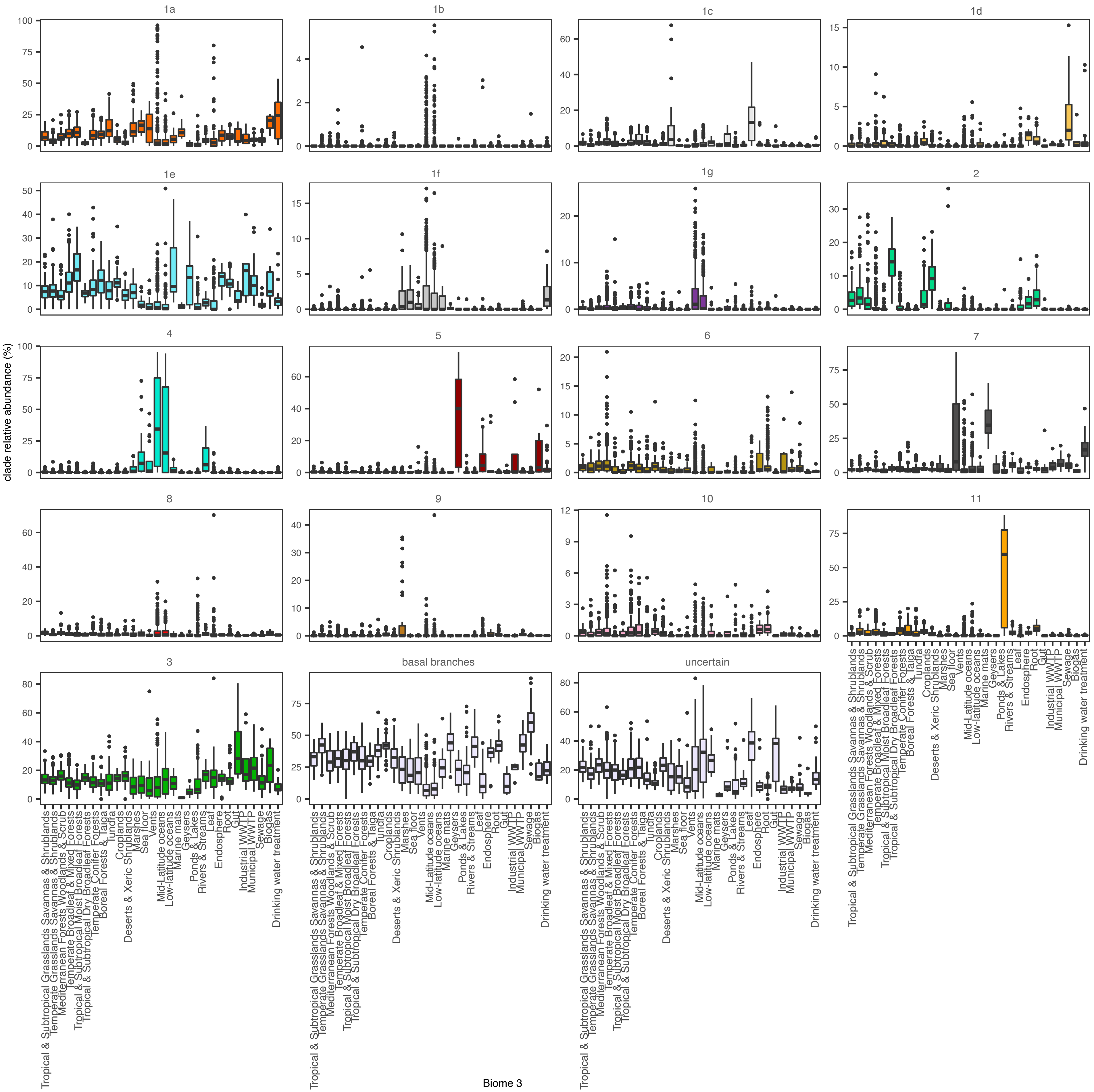

1a
1b
1c
1d
1e
1f
1g
2
4
5
6
7
8
9
10
11
3
basal branches
uncertain
clade relative abundance (%)
Biome 3
Tropical & Subtropical Grasslands Savannas & Shrublands
Temperate Grasslands Savannas & Shrublands
Mediterranean Forests Woodlands & Scrub
Temperate Broadleaf & Mixed Forests
Tropical & Subtropical Moist Broadleaf Forests
Tropical & Subtropical Dry Broadleaf Forests
Temperate Conifer Forests
Boreal Forests & Taiga
Tundra
Croplands
Deserts & Xeric Shrublands
Marshes
Sea floor
Vents
Mid-Latitude oceans
Low-latitude oceans
Marine mats
Geysers
Ponds & Lakes
Rivers & Streams
Leaf
Endosphere
Root
Gut
Industrial WWTP
Municipal WWTP
Sewage
Biogas
Drinking water treatment

Fig. S4 Relative abundance of *nirK* clades in metagenomes from various biomes. Basal branches indicate the best placement for a read occurred in regions of the phylogeny not belonging to a specific clade. Unknown indicates best placements was distributed across multiple clades. Remaining clades follow Fig. 1. Boxplots show median and quartiles, and whiskers show 95 percentiles, and values outside 95 percentiles are shown as points. Please note differences in y-axis scale across plots.
