# Supplementary material for: Phylogenetics and environmental distribution of nitric oxide-forming nitrite reductases reveal their distinct functional and ecological roles
Source: ISME Commun. 2024 Feb 2;4(1):ycae020. doi: 10.1093/ismeco/ycae020 (PMC10999283; doi:10.1093/ismeco/ycae020)
Supplement: Supplementary_File_1_ycae020 [file supplementary_file_1_ycae020.pdf]

Phylogenetics and environmental distribution of nitric oxide forming nitrite reductases reveals their distinct functional and ecological roles

Authors: Grace Pold, Germán Bonilla-Rosso, Aurélien Saghaï, Marc Strous, Christopher M. Jones, Sara Hallin

Supplementary Materials and Methods

Naming clades:

Major clades in each tree were first delineated based on groupings identified in previous literature [1,2], reserving clade 1 in each protein for the "canonical clade", and letters were used within each larger clade to delineate sub clades. Primarily structural features and secondarily taxonomy and ecological traits were used to refine clade delineation. Clades were defined as deeply as possible on the phylogeny within these constraints, leading to statistically supported clades of variable phylogenetic depth. In some instances this meant generating new clades from previously proposed clades that were not well-supported in the present phylogeny. Thus, clades with similar naming number-letter hierarchy level do not necessarily originate at similar depths on the phylogeny, and we refer to these well-supported groups of sequences in the phylogeny as clades independent of number-letter hierarchy.

Exclusion of halophilic archaea and *Pyrobaculum* NirS

Functional NirS has also been demonstrated in *Pyrobaculum* species (Thermoproteota [3,4]. Despite carrying all the conserved motifs, we excluded these *Pyrobaculum* sequences from our phylogeny because the haem d_1 and cyt_c domains are encoded in opposite directions in

the genome, which led to an unresolvable long branch upon re-orienting and concatenating them.

Halobacteriota NirS-like proteins were included as an outgroup and excluded from *nirS* counts in the metagenome survey because they lack the first two characteristic motifs corresponding to the *cyt*$_c$ domain. Using the previously described search for genes encoding enzymes involved in NirS assembly combined with genome viewer in NCBI to look for potential alternative heme assembly proteins [3] and *cyt*$_c$ domain-containing motifs, we confirmed the absence of the evidence that these proteins are *cyt*$_{cd}$ NirS. An additional reason for excluding this clade from *nirS* gene fragments counts is that absence of the *cyt*$_c$ domain led to strange behaviour of the search and place algorithm. An excess of *nirS* reads were placed in this gapped region of the alignment and subsequently annotated as haloarchaeal, despite being derived from habitats such as forest soils where Halobacteriota are rare, and where Halobacteriota *nirK* reads were below detection. Furthermore, BLAST of a subset (n=20) of the reads annotated as haloarchaeal *nirS* but mapped in their entirety to this 75aa gap in the beginning of the alignment against the UniProt database indicated that none of them most closely matched archaea; instead, most (18; 90%) mapped as non-nitrite reductase bacterial cytochrome C or cytochrome C oxidase, often with at least 60% identity (13; 65%). By contrast, short reads from this N-terminal region which GraftM placed in the canonical *nirS* portion of the tree correctly mapped to proteobacterial nitrite reductase or cytochrome C.

Structural features and nitrite reductase helper genes

We searched for *nirF, nirN, nirJ, nirE, nirB* and *nirT* in assemblies carrying *nirS,* and *nirV* in assemblies carrying *nirK.* The seed alignments for NirJ (TIGR04051, TIGR04055, TIGR04054) and NirE (cd11642) were derived from the NCBI's Conserved Domains Database. Seed alignments for NirF and NirN were derived from the original NirS search,

and were readily differentiated from NirS using a phylogenetic approach. The HMMs for NirB (UniProt P24037), NirT (UniProt P24038) and NirV (NCBI AAK08123.1) were generated using protein BLAST searches with the aforementioned reference sequences against NCBI's ClusteredNR database [5]. We stochastically selected a subset of the 1000 top hits to be aligned and exported using the multiple alignment function accessible from the BLAST outputs, and then checked if the sequences were aligned at the important ligands and catalytic residues in ARB [6]. We searched for the structural features TAT, lipobox, and Sec-type signal peptides using the online version of SignalP 6.0 [7] and transmembrane domains using DeepTMHMM [8], both with default settings. Clade-specific insertions and deletions[9], and cytochrome C ($-CX_2CHX_{50}M-$) and cupredoxin ($CX_{2\text{-}4}HX_{2\text{-}4}M$) motifs in the C and N termini of the proteins were identified in ARB [6].

Inferring redox traits from protein-coding gene composition

We looked for nitric oxide reductases within the heme-copper oxidase (HCO) superfamily using the same method used for identifying NirK and NirS, but used the database generated by Murali and colleagues as a starting seed for our hmmsearch [10,11]. Genes were categorized into classes of *nor* also following Murali *et al.* [10,11]. We identified nitrous oxide reductases (*nosZ*) similarly, but used the reference database from Graf *et al.* 2014 as our seed; hydrogenases associated with hydrogen oxidation (group 1, 2a) and reduction (group 4 b,c,e) using the alignment in [12]; nitrite oxidation using the alignment and structural information provided by [13]; ammonium (*amoA*) and methane (*pmoA*) oxidation against the database provided by [14]; and sulfur compound oxidation (*sox*, ox-*apr*, ox-*dsr*, *sat*, *shdr*, or *sor*) and reduction (APS sulfate reduction: *apr*AB, *qmo*ABC; sulfite reduction: *dsrABCDNTMK, mccABCD, AsrABC*; tetrathionate reduction: *Ttr*ABC) using HMMs and gene neighborhood information provided in [15]. The capacity for anammox (*hzoA,hzsA*),

methane oxidation (*mmoB*/*dmpM*, *pmoABC*), iron (*fmnA*/*dmkA*/*fmnB*/*pplA*/*ndh2*/*eetAB*/*dmkB*, *mtrABC*), selenate (*ygfKMN*), nitrate (*napAB*, *narGH*) and dissimilatory arsenate reduction (*arrA*), and iron (*cyc123; foxABCXYZ*), nitrite (*nxrAB*) and manganese oxidation (*mnxG*) were predicted using HMMs and gene neighborhood information from FeGenie and Lithogenie [16]. *nxrA*/*napA* and *amoA*/*pmoA*/*hcoA* were further checked by assessing the alignment and constructing a phylogeny using FastTree, and additional taxonomy-based verification. The capacity for reductive dehalogenation was evaluated using HMMs built from the Reductive Dehalogenase Database [17]; sequences lying within known reductive dehalogenase diversity in a combined FastTree phylogeny were kept. The complete denitrification trait was defined as having *nir*, *nor*, and *nosZ* genes as depicted below.

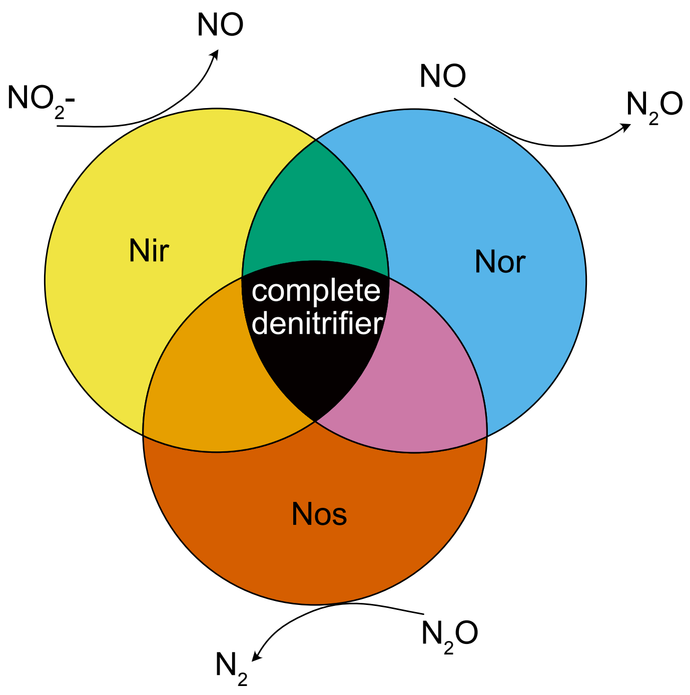

Supplementary methods figure 1: complete denitrification refers to the potential to transform nitrite into dinitrogen via NO (completed by Nir), N_2O (Nor) and finally N_2 (Nos). Incomplete denitrification in organisms encoding NirK and/or NirS refers to the presence of Nir+Nor (green), Nir+Nos (orange), or the presence of just Nir (yellow).

References:

1. Wei W, Isobe K, Nishizawa T *et al.* Higher diversity and abundance of denitrifying microorganisms in environments than considered previously. *ISME J* 2015;**9**:1954–65.

2. Bonilla-Rosso G, Wittorf L, Jones CM *et al.* Design and evaluation of primers targeting genes encoding NO-forming nitrite reductases: implications for ecological inference of denitrifying communities. *Sci Rep* 2016;**6**:39208.

3. Storbeck S, Rolfes S, Raux-Deery E *et al.* A Novel Pathway for the Biosynthesis of Heme in Archaea: Genome-Based Bioinformatic Predictions and Experimental Evidence. *Archaea* 2010;**2010**:e175050.

4. Feinberg LF, Holden JF. Characterization of dissimilatory Fe(III) versus NO3- reduction in the hyperthermophilic archaeon Pyrobaculum aerophilum. *J Bacteriol* 2006;**188**:525–31.

5. Staff N. New ClusteredNR database: faster searches and more informative BLAST results. *NCBI Insights* 2022. https://ncbiinsights.ncbi.nlm.nih.gov/2022/05/02/clusterednr_1/

6. Westram R, Bader K, Preusse E *et al.* ARB: a software environment for sequence data. *Handbook of Molecular Microbial Ecology I: Metagenomics and Complementary Approaches*. Wiley, 399–406.

7. Teufel F, Almagro Armenteros JJ, Johansen AR *et al.* SignalP 6.0 predicts all five types of signal peptides using protein language models. *Nat Biotechnol* 2022;**40**:1023–5.

8. Hallgren J, Tsirigos KD, Pedersen MD *et al.* DeepTMHMM predicts alpha and beta transmembrane proteins using deep neural networks. 2022:2022.04.08.487609.

9. Decleyre H, Heylen F, Bjorn T *et al.* Highly diverse nirK genes comprise two major clades that harbour ammonium-producing denitrifiers. *BMC Genomics* 2016;**17**:155.

10. Murali R, Pace LA, Sanford RA *et al.* Diversity and evolution of nitric oxide reduction. 2021:2021.10.15.464467.

11. Murali R, Hemp J, Gennis RB. Evolution of quinol oxidation within the heme-copper oxidoreductase superfamily. *Biochim Biophys Acta BBA - Bioenerg* 2022:148907.

12. Greening C, Biswas A, Carere CR *et al.* Genomic and metagenomic surveys of hydrogenase distribution indicate H2 is a widely utilised energy source for microbial growth and survival. *ISME J* 2016;**10**:761–77.

13. Chicano TM, Dietrich L, de Almeida NM *et al.* Structural and functional characterization of the intracellular filament-forming nitrite oxidoreductase multiprotein complex. *Nat Microbiol* 2021;**6**:1129–39.

14. Diamond S, Lavy A, Crits-Christoph A *et al.* Soils and sediments host Thermoplasmata archaea encoding novel copper membrane monooxygenases (CuMMOs). *ISME J* 2022;**16**:1348–62.

15. Tanabe TS, Dahl C. HMS-S-S: A tool for the identification of Sulphur metabolism-related genes and analysis of operon structures in genome and metagenome assemblies. *Mol Ecol Resour* 2022;**22**:2758–74.

16. Garber AI, Nealson KH, Okamoto A *et al.* FeGenie: a comprehensive tool for the identification of iron genes and iron gene neighborhoods in genome and metagenome assemblies. *Front Microbiol* 2020;**11**, DOI: doi.org/10.3389/fmicb.2020.00037.

17. Molenda O, Jácome LAP, Cao X *et al.* Insights into origins and function of the unexplored majority of the reductive dehalogenase gene family as a result of genome assembly and ortholog group classification. *Environ Sci Process Impacts* 2020;**22**:663–78.

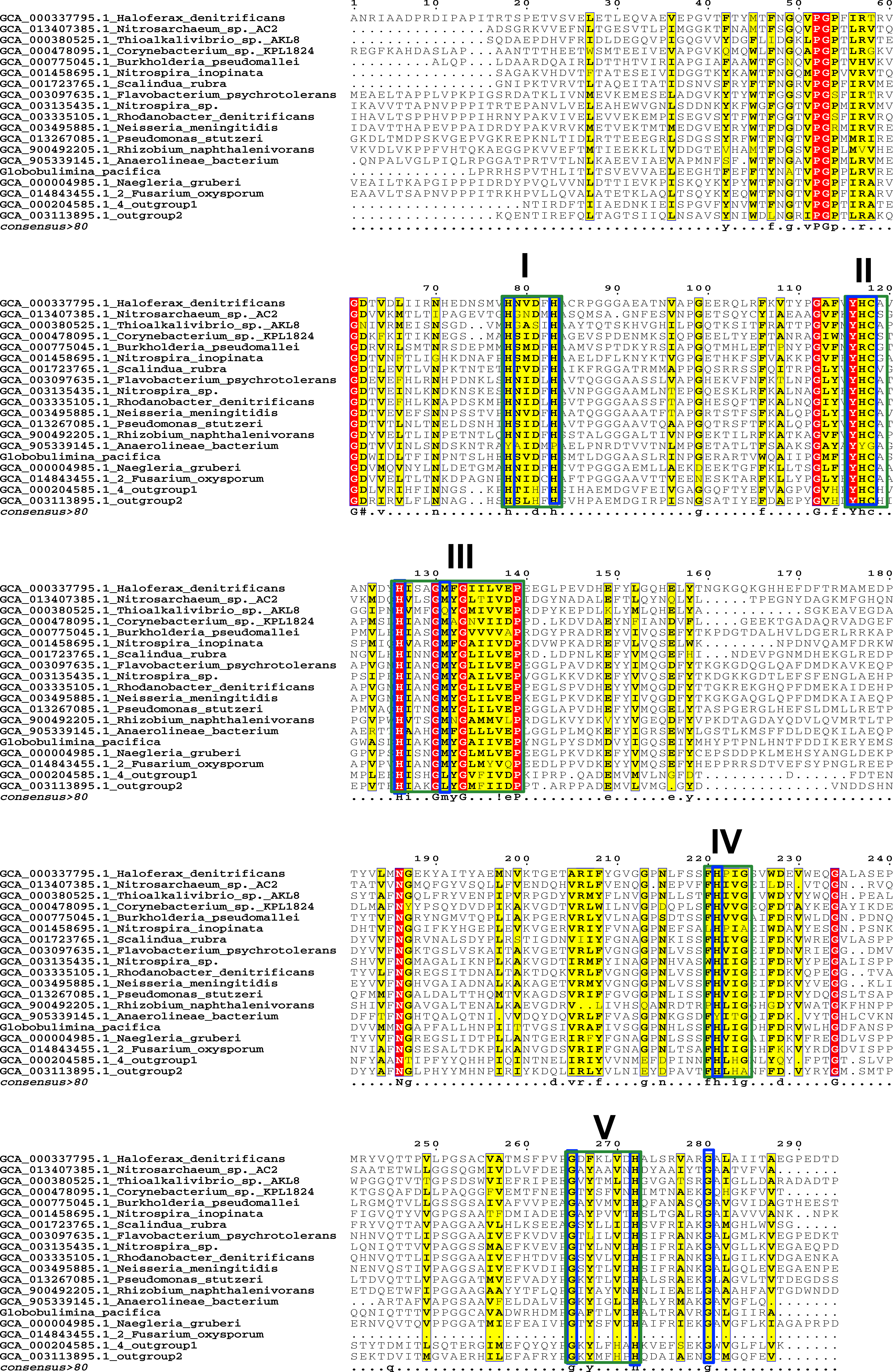
GCA_000337795.1_Haloferax_denitrificans
GCA_013407385.1_Nitrosarchaeum_sp._AC2
GCA_000380525.1_Thioalkalivibrio_sp._AKL8
GCA_000478095.1_Corynebacterium_sp._KPL1824
GCA_000775045.1_Burkholderia_pseudomallei
GCA_001458695.1_Nitrospira_inopinata
GCA_001723765.1_Scalindua_rubra
GCA_003097635.1_Flavobacterium_psychrotolerans
GCA_003135435.1_Nitrospira_sp.
GCA_003335105.1_Rhodanobacter_denitrificans
GCA_003495885.1_Neisseria_meningitidis
GCA_013267085.1_Pseudomonas_stutzeri
GCA_900492205.1_Rhizobium_naphthalenivorans
GCA_905339145.1_Anaerolineae_bacterium
Globobulimina_pacifica
GCA_000004985.1_Naegleria_gruberi
GCA_014843455.1_2_Fusarium_oxysporum
GCA_000204585.1_4_outgroup1
GCA_003113895.1_outgroup2
consensus>80
I
II
III
IV
V

Fig. S1 Representative NirS alignment. Yellow boxes represent >70% conserved residues and red boxes represent identical residues. Numbered red boxes mark characteristic conserved motifs (I, III, IV, V, VII and IV distinguish NirS from NirN and NirF) and blue boxes mark functionally important residues, and asterisks beneath alignment mark specific residues where nirS can be differentiated from NirF and NirN. Notably, motif I on the cyt_c domain was generally characterised by -Ca**G**CHg- in NirS, motif VI by -LHD- (vs. -PyD- in NirF and -LDD- in NirN), and motif VII at the cyt_{d1} domain is identified by the universal absence of a proline in the third position in NirN and NirF (i.e. -PHpGpG- vs. PH!PgeG). Clade 1c: *Hydrogenophilales sp.;* clade 3: *Sulfurimonas paralvinellae;* clade 4: *Calidifontibacillus erzurumensis;* clade 1d: *Brocadia sp. WS118;* clade 1e: *c. Magnetaquicoccus inordinatus;* clade 1a: *Stutzerimonas stutzeri;* clade 1f: *Deltaproteobacteria bact. GWA2_43_19;* clade 5: *Levilinea saccharolytica;* clade 1h: *Scalindua brodae;* clade 1j: *Thermus oshimai;* clade 1b: *Thauera linaloolentis;* clade 2: *Methylomicrobium album;* clade 6: candidatus *Methylomirabilis oxyfera;* clade 1g: *Roseiflexus castenholzii;* clade 1k: *Colwellia psychrerythraea;* halophilic archaea NirS-like: *Natrinema pellirubrum.* The Fig. was prepared in ESPscript/ENDscript.

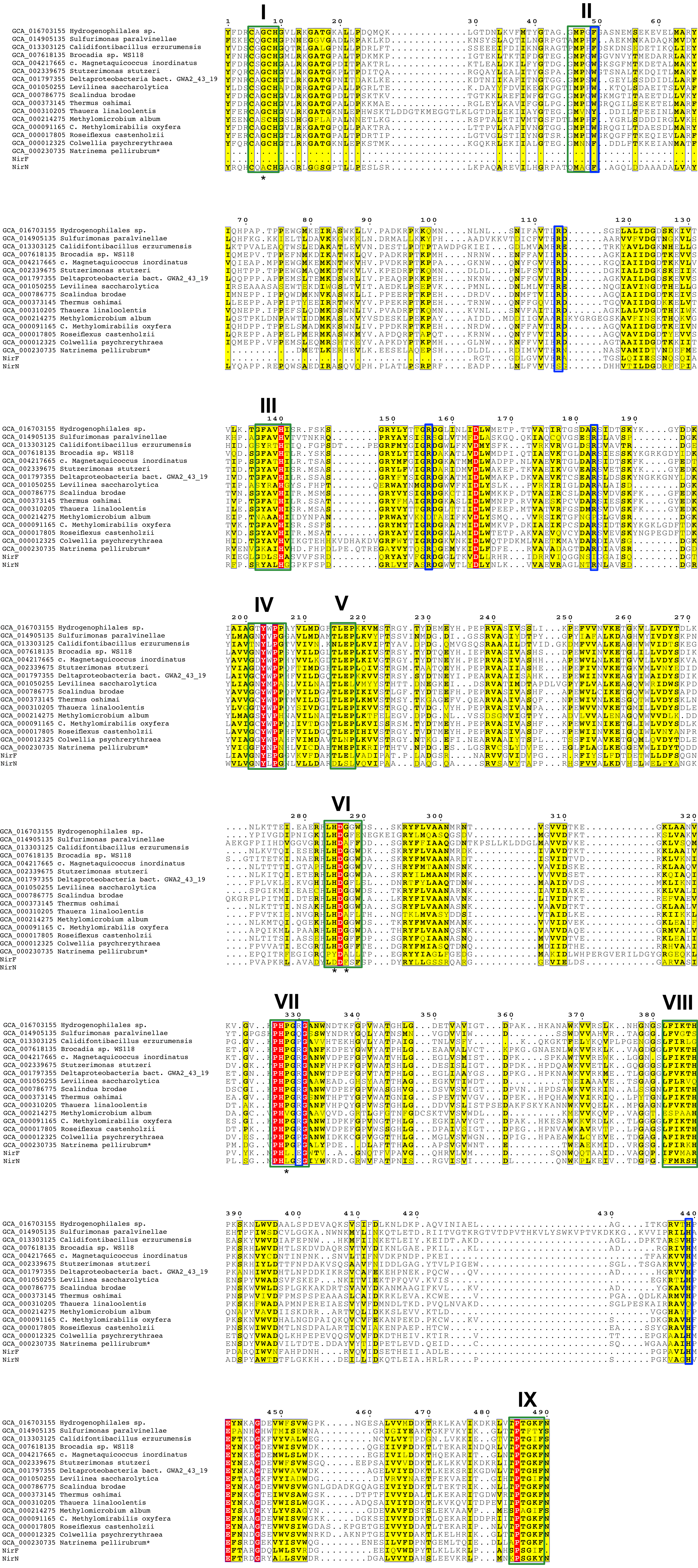
I
II
III
IV
V
VI
VII
VIII
IX
GCA_016703155 Hydrogenophilales sp.
GCA_014905135 Sulfurimonas paralvinellae
GCA_013303125 Calidifontibacillus erzurumensis
GCA_007618135 Brocadia sp. WS118
GCA_004217665 c. Magnetaquicoccus inordinatus
GCA_002339675 Stutzerimonas stutzeri
GCA_001797355 Deltaproteobacteria bact. GWA2_43_19
GCA_001050255 Levilinea saccharolytica
GCA_000786775 Scalindua brodae
GCA_000373145 Thermus oshimai
GCA_000310205 Thauera linaloolentis
GCA_000214275 Methylomicrobium album
GCA_000091165 C. Methylomirabilis oxyfera
GCA_000017805 Roseiflexus castenholzii
GCA_000012325 Colwellia psychrerythraea
GCA_000230735 Natrinema pellirubrum*
NirF
NirN

Fig. S2 Representative NirK alignment. Yellow boxes represent >70% conserved residues and red boxes represent identical residues. Green boxes mark characteristic conserved motifs differentiating NirK from other MCOs (**H**niDf**H**- (or -**H**ni**S**f**H**- in clade 5; [91]) rather than **H**ti**H**F**H**- in motif I; Y**H**Cap- instead of -Y**H**C**H**vm- in motif II; -trp**H**vIG- instead of -insf**H**l**H**G- in motif IV; and -GiYAyd**H**- instead of GkYmF**H**A**H**- in motif V), and blue boxes mark functionally important residues. From top to bottom, the sequences represent: clade 1c: *Haloferax denitrificans*; clade 4: *Nitrosoarcheum sp.;* clade 5: *Thioalkalivibrio thiocyanoxidans*; clade 4: *Nitrososphaera viennensis*; Clade 7: *Nitrospira nitrificans*; clade 1g: *Fusarium oxysporum;* cladeless anammox: *Scalindua rubra;* clade 8: *Paenibacillus amylolyticus;* clade 3: *Propionibacterium australiense*; clade 11: *Burkholderia mallei;* clade 1a: *Nitrosomonas oligotropha*; clade 9: *Rhodanobacter glycinis*. The Fig. was prepared in ESPscript/ENDscript.

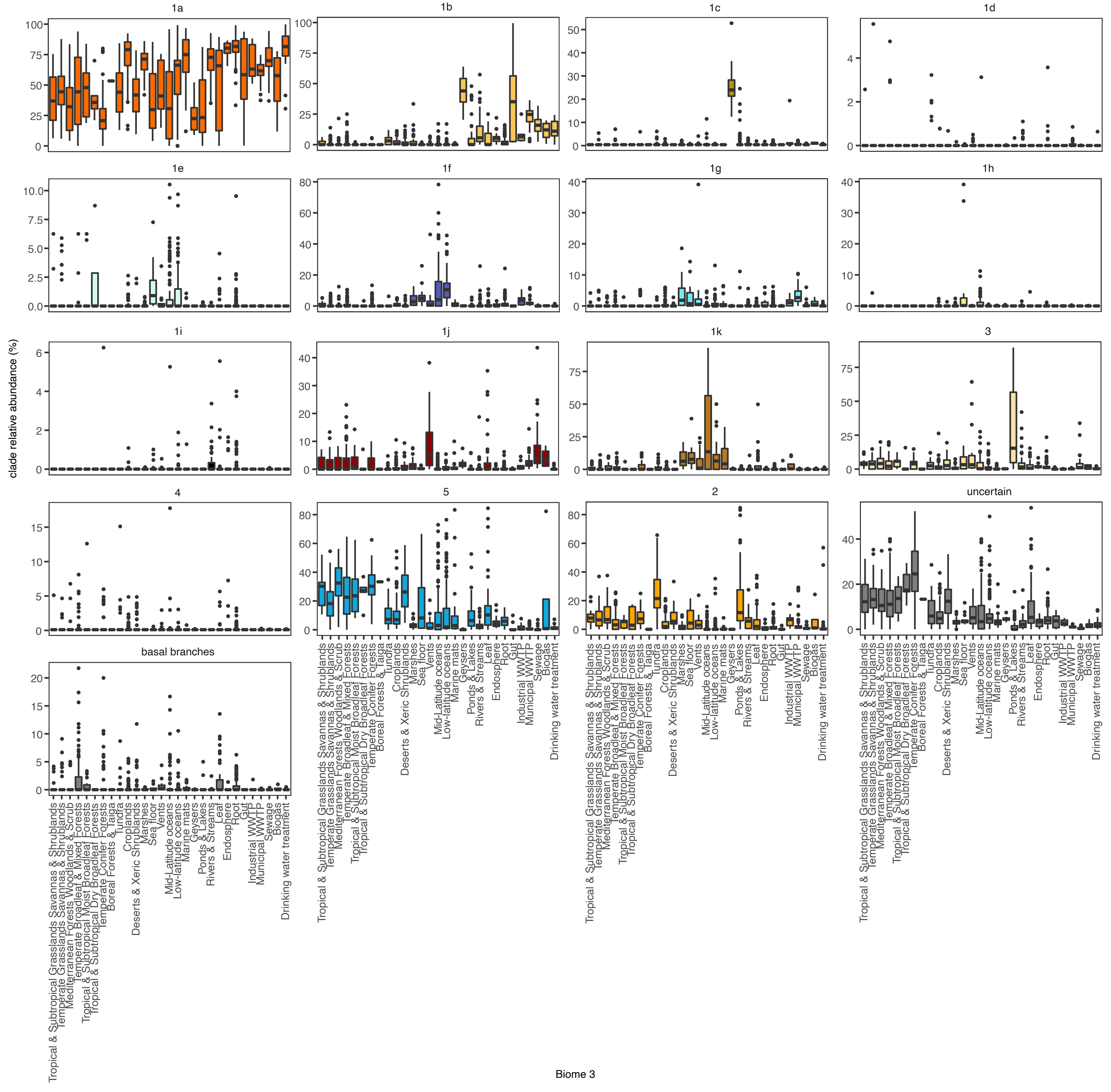
1a
1b
1c
1d
1e
1f
1g
1h
1i
1j
1k
3
4
5
2
uncertain
basal branches
clade relative abundance (%)
Biome 3
Tropical & Subtropical Grasslands Savannas & Shrublands
Temperate Grasslands Savannas & Shrublands
Mediterranean Forests Woodlands & Scrub
Temperate Broadleaf & Mixed Forests
Tropical & Subtropical Moist Broadleaf Forests
Tropical & Subtropical Dry Broadleaf Forests
Temperate Conifer Forests
Boreal Forests & Taiga
Tundra
Croplands
Deserts & Xeric Shrublands
Marshes
Sea floor
Vents
Mid-Latitude oceans
Low-latitude oceans
Marine mats
Geysers
Ponds & Lakes
Rivers & Streams
Leaf
Endosphere
Root
Gut
Industrial WWTP
Municipal WWTP
Sewage
Biogas
Drinking water treatment

Fig. S3 Relative abundance of *nirS* clades in metagenomes from various biomes. Basal branches indicate the best placement for a read occurred in regions of the phylogeny not belonging to a specific clade. Unknown indicates best placements was distributed across multiple clades. Remaining clades follow Fig. 2. Boxplots show median and quartiles, and whiskers show 95 percentiles, and values outside 95 percentiles are shown as points. Please note differences in y-axis scale across plots.

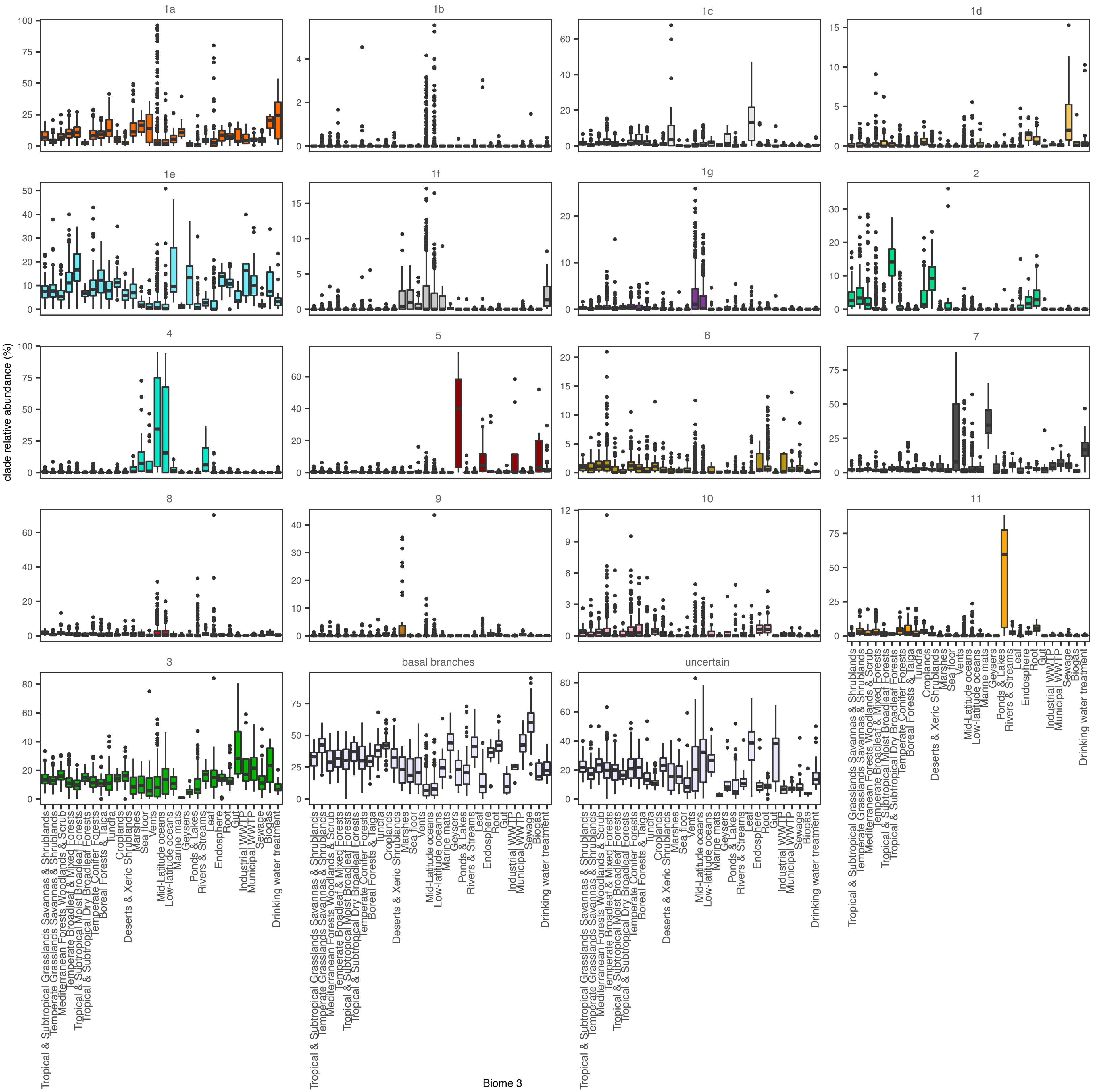
1a
1b
1c
1d
1e
1f
1g
2
4
5
6
7
8
9
10
11
3
basal branches
uncertain
clade relative abundance (%)
Biome 3
Tropical & Subtropical Grasslands Savannas & Shrublands
Temperate Grasslands Savannas & Shrublands
Mediterranean Forests Woodlands & Scrub
Temperate Broadleaf & Mixed Forests
Tropical & Subtropical Moist Broadleaf Forests
Tropical & Subtropical Dry Broadleaf Forests
Temperate Conifer Forests
Boreal Forests & Taiga
Tundra
Croplands
Deserts & Xeric Shrublands
Marshes
Sea floor
Vents
Mid-Latitude oceans
Low-latitude oceans
Marine mats
Geysers
Ponds & Lakes
Rivers & Streams
Leaf
Endosphere
Root
Gut
Industrial WWTP
Municipal WWTP
Sewage
Biogas
Drinking water treatment

Fig. S4 Relative abundance of *nirK* clades in metagenomes from various biomes. Basal branches indicate the best placement for a read occurred in regions of the phylogeny not belonging to a specific clade. Unknown indicates best placements was distributed across multiple clades. Remaining clades follow Fig. 1. Boxplots show median and quartiles, and whiskers show 95 percentiles, and values outside 95 percentiles are shown as points. Please note differences in y-axis scale across plots.
