# Supplementary material for: Phylogenetics and environmental distribution of nitric oxide-forming nitrite reductases reveal their distinct functional and ecological roles
Source: ISME Commun. 2024 Feb 2;4(1):ycae020. doi: 10.1093/ismeco/ycae020 (PMC10999283; doi:10.1093/ismeco/ycae020)
Supplement: Supplementary_Materials_and_Methods_ycae020 [file supplementary_materials_and_methods_ycae020.pdf]

Phylogenetics and environmental distribution of nitric oxide forming nitrite reductases reveals their distinct functional and ecological roles

Authors: Grace Pold, Germán Bonilla-Rosso, Aurélien Saghaï, Marc Strous, Christopher M. Jones, Sara Hallin

Supplementary Materials and Methods

Naming clades:

Major clades in each tree were first delineated based on groupings identified in previous literature [1,2], reserving clade 1 in each protein for the "canonical clade", and letters were used within each larger clade to delineate sub clades. Primarily structural features and secondarily taxonomy and ecological traits were used to refine clade delineation. Clades were defined as deeply as possible on the phylogeny within these constraints, leading to statistically supported clades of variable phylogenetic depth. In some instances this meant generating new clades from previously proposed clades that were not well-supported in the present phylogeny. Thus, clades with similar naming number-letter hierarchy level do not necessarily originate at similar depths on the phylogeny, and we refer to these well-supported groups of sequences in the phylogeny as clades independent of number-letter hierarchy.

Exclusion of halophilic archaea and *Pyrobaculum* NirS

Functional NirS has also been demonstrated in *Pyrobaculum* species (Thermoproteota [3,4]. Despite carrying all the conserved motifs, we excluded these *Pyrobaculum* sequences from our phylogeny because the haem d_1 and *cyt*$_c$ domains are encoded in opposite directions in

the genome, which led to an unresolvable long branch upon re-orienting and concatenating them.

Halobacteriota NirS-like proteins were included as an outgroup and excluded from *nirS* counts in the metagenome survey because they lack the first two characteristic motifs corresponding to the *cyt*$_c$ domain. Using the previously described search for genes encoding enzymes involved in NirS assembly combined with genome viewer in NCBI to look for potential alternative heme assembly proteins [3] and *cyt*$_c$ domain-containing motifs, we confirmed the absence of the evidence that these proteins are *cyt*$_{cd}$ NirS. An additional reason for excluding this clade from *nirS* gene fragments counts is that absence of the *cyt*$_c$ domain led to strange behaviour of the search and place algorithm. An excess of *nirS* reads were placed in this gapped region of the alignment and subsequently annotated as haloarchaeal, despite being derived from habitats such as forest soils where Halobacteriota are rare, and where Halobacteriota *nirK* reads were below detection. Furthermore, BLAST of a subset (n=20) of the reads annotated as haloarchaeal *nirS* but mapped in their entirety to this 75aa gap in the beginning of the alignment against the UniProt database indicated that none of them most closely matched archaea; instead, most (18; 90%) mapped as non-nitrite reductase bacterial cytochrome C or cytochrome C oxidase, often with at least 60% identity (13; 65%). By contrast, short reads from this N-terminal region which GraftM placed in the canonical *nirS* portion of the tree correctly mapped to proteobacterial nitrite reductase or cytochrome C.

Structural features and nitrite reductase helper genes

We searched for *nirF, nirN, nirJ, nirE, nirB* and *nirT* in assemblies carrying *nirS,* and *nirV* in assemblies carrying *nirK*. The seed alignments for NirJ (TIGR04051, TIGR04055, TIGR04054) and NirE (cd11642) were derived from the NCBI's Conserved Domains Database. Seed alignments for NirF and NirN were derived from the original NirS search,

and were readily differentiated from NirS using a phylogenetic approach. The HMMs for NirB (UniProt P24037), NirT (UniProt P24038) and NirV (NCBI AAK08123.1) were generated using protein BLAST searches with the aforementioned reference sequences against NCBI's ClusteredNR database [5]. We stochastically selected a subset of the 1000 top hits to be aligned and exported using the multiple alignment function accessible from the BLAST outputs, and then checked if the sequences were aligned at the important ligands and catalytic residues in ARB [6]. We searched for the structural features TAT, lipobox, and Sec-type signal peptides using the online version of SignalP 6.0 [7] and transmembrane domains using DeepTMHMM [8], both with default settings. Clade-specific insertions and deletions[9], and cytochrome C (-$CX_2CHX_{50}M$-) and cupredoxin ($CX_{2\text{-}4}HX_{2\text{-}4}M$) motifs in the C and N termini of the proteins were identified in ARB [6].

Inferring redox traits from protein-coding gene composition

We looked for nitric oxide reductases within the heme-copper oxidase (HCO) superfamily using the same method used for identifying NirK and NirS, but used the database generated by Murali and colleagues as a starting seed for our hmmsearch [10,11]. Genes were categorized into classes of *nor* also following Murali *et al.* [10,11]. We identified nitrous oxide reductases (*nosZ*) similarly, but used the reference database from Graf *et al.* 2014 as our seed; hydrogenases associated with hydrogen oxidation (group 1, 2a) and reduction (group 4 b,c,e) using the alignment in [12]; nitrite oxidation using the alignment and structural information provided by [13]; ammonium (*amoA*) and methane (*pmoA*) oxidation against the database provided by [14]; and sulfur compound oxidation (*sox*, ox-*apr*, ox-*dsr*, *sat*, *shdr*, or *sor*) and reduction (APS sulfate reduction: *apr*AB, *qmo*ABC; sulfite reduction: *dsrABCDNTMK, mccABCD, AsrABC*; tetrathionate reduction: *Ttr*ABC) using HMMs and gene neighborhood information provided in [15]. The capacity for anammox (*hzoA,hzsA*),

methane oxidation (*mmoB*/*dmpM*, *pmoABC*), iron (*fmnA*/*dmkA*/*fmnB*/*pplA*/*ndh2*/*eetAB*/*dmkB*, *mtrABC*), selenate (*ygfKMN*), nitrate (*napAB*, *narGH*) and dissimilatory arsenate reduction (*arrA*), and iron (*cyc123; foxABCXYZ*), nitrite (*nxrAB*) and manganese oxidation (*mnxG*) were predicted using HMMs and gene neighborhood information from FeGenie and Lithogenie [16]. *nxrA*/*napA* and *amoA*/*pmoA*/*hcoA* were further checked by assessing the alignment and constructing a phylogeny using FastTree, and additional taxonomy-based verification. The capacity for reductive dehalogenation was evaluated using HMMs built from the Reductive Dehalogenase Database [17]; sequences lying within known reductive dehalogenase diversity in a combined FastTree phylogeny were kept. The complete denitrification trait was defined as having *nir*, *nor*, and *nosZ* genes as depicted below.

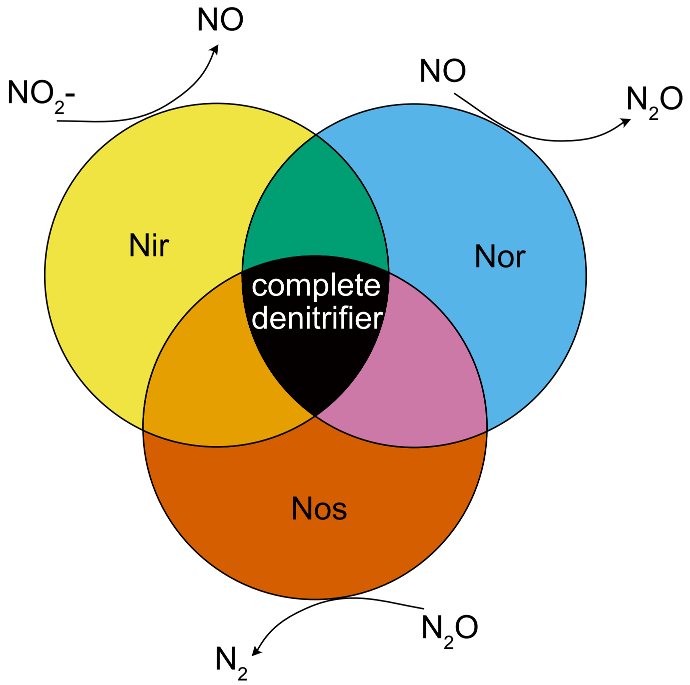

Supplementary methods figure 1: complete denitrification refers to the potential to transform nitrite into dinitrogen via NO (completed by Nir), N_2O (Nor) and finally N_2 (Nos). Incomplete denitrification in organisms encoding NirK and/or NirS refers to the presence of Nir+Nor (green), Nir+Nos (orange), or the presence of just Nir (yellow).

References:

1. Wei W, Isobe K, Nishizawa T *et al.* Higher diversity and abundance of denitrifying microorganisms in environments than considered previously. *ISME J* 2015;**9**:1954–65.

2. Bonilla-Rosso G, Wittorf L, Jones CM *et al.* Design and evaluation of primers targeting genes encoding NO-forming nitrite reductases: implications for ecological inference of denitrifying communities. *Sci Rep* 2016;**6**:39208.

3. Storbeck S, Rolfes S, Raux-Deery E *et al.* A Novel Pathway for the Biosynthesis of Heme in Archaea: Genome-Based Bioinformatic Predictions and Experimental Evidence. *Archaea* 2010;**2010**:e175050.

4. Feinberg LF, Holden JF. Characterization of dissimilatory Fe(III) versus NO3- reduction in the hyperthermophilic archaeon Pyrobaculum aerophilum. *J Bacteriol* 2006;**188**:525–31.

5. Staff N. New ClusteredNR database: faster searches and more informative BLAST results. *NCBI Insights* 2022. https://ncbiinsights.ncbi.nlm.nih.gov/2022/05/02/clusterednr_1/

6. Westram R, Bader K, Preusse E *et al.* ARB: a software environment for sequence data. *Handbook of Molecular Microbial Ecology I: Metagenomics and Complementary Approaches*. Wiley, 399–406.

7. Teufel F, Almagro Armenteros JJ, Johansen AR *et al.* SignalP 6.0 predicts all five types of signal peptides using protein language models. *Nat Biotechnol* 2022;**40**:1023–5.

8. Hallgren J, Tsirigos KD, Pedersen MD *et al.* DeepTMHMM predicts alpha and beta transmembrane proteins using deep neural networks. 2022:2022.04.08.487609.

9. Decleyre H, Heylen F, Bjorn T *et al.* Highly diverse nirK genes comprise two major clades that harbour ammonium-producing denitrifiers. *BMC Genomics* 2016;**17**:155.

10. Murali R, Pace LA, Sanford RA *et al.* Diversity and evolution of nitric oxide reduction. 2021:2021.10.15.464467.

11. Murali R, Hemp J, Gennis RB. Evolution of quinol oxidation within the heme-copper oxidoreductase superfamily. *Biochim Biophys Acta BBA - Bioenerg* 2022:148907.

12. Greening C, Biswas A, Carere CR *et al.* Genomic and metagenomic surveys of hydrogenase distribution indicate H2 is a widely utilised energy source for microbial growth and survival. *ISME J* 2016;**10**:761–77.

13. Chicano TM, Dietrich L, de Almeida NM *et al.* Structural and functional characterization of the intracellular filament-forming nitrite oxidoreductase multiprotein complex. *Nat Microbiol* 2021;**6**:1129–39.

14. Diamond S, Lavy A, Crits-Christoph A *et al.* Soils and sediments host Thermoplasmata archaea encoding novel copper membrane monooxygenases (CuMMOs). *ISME J* 2022;**16**:1348–62.

15. Tanabe TS, Dahl C. HMS-S-S: A tool for the identification of Sulphur metabolism-related genes and analysis of operon structures in genome and metagenome assemblies. *Mol Ecol Resour* 2022;**22**:2758–74.

16. Garber AI, Nealson KH, Okamoto A *et al.* FeGenie: a comprehensive tool for the identification of iron genes and iron gene neighborhoods in genome and metagenome assemblies. *Front Microbiol* 2020;**11**, DOI: doi.org/10.3389/fmicb.2020.00037.

17. Molenda O, Jácome LAP, Cao X *et al.* Insights into origins and function of the unexplored majority of the reductive dehalogenase gene family as a result of genome assembly and ortholog group classification. *Environ Sci Process Impacts* 2020;**22**:663–78.
